# M2 macrophage-secreted KYNU promotes stemness remodeling and malignant behavior in endometrial cancer via the SOD2-mtROS-ERO1α-UPR^ER^ axis

**DOI:** 10.1186/s13046-025-03285-y

**Published:** 2025-07-04

**Authors:** Xin Pan, Wantong Wang, Yuting Wang, JiaHui Gu, Xiaoxin Ma

**Affiliations:** Department of Obstetrics and Gynecology, Shengjing Hospital of China Medical University, 39 Huaxiang Road, Tiexi District, Shenyang City, 110022 Liaoning Province China

**Keywords:** M2 macrophage, Endometrial cancer, KYNU, Tumor microenvironment; endoplasmic reticulum, Tumorigenesis

## Abstract

**Background:**

M2 macrophages are known to be involved in tumorigenesis. However, the mechanism by which they promote tumor progression in endometrial cancer (EC) remains largely unknown. Kynureninase (KYNU) has been found to be associated with the progression of various tumors, but research on endometrium is limited to embryo transfer. Therefore, a better understanding of KYNU as a potential therapeutic target in EC treatment is needed. This study aimed to elucidate the mechanism by which M2 macrophage-secreted KYNU influences the malignant biological and stemness remodeling of EC via the SOD2-mtROS-ERO1α and endoplasmic reticulum unfolded protein response (UPR^ER^) pathway.

**Methods:**

We used flow cytometry for cell sorting. Fluorescence experiments were conducted to reveal spatial position of protein, and. Western blot and qRT‒PCR were used to detect the protein and mRNA levels, respectively. The interaction between KYNU and superoxide dismutase 2 (SOD2) was demonstrated using coimmunoprecipitation experiments. Furthermore, the mechanism between activating transcription factor 4 (ATF4) and the KYNU was assessed using chromatin immunoprecipitation and dual luciferase assays. Cell Counting Kit-8, flow cytometry, and transwell assays were used to detect tumor cell proliferation, apoptosis, and invasion capacities. Student’s t test and one-way analysis of variance (ANOVA) were used to compare groups.

**Results:**

M2 macrophage-secreted KYNU induced malignant behavior and stemness via the SOD2-mtROS-ERO1α-UPR^ER^ pathway, contributing to a positive feedback loop for tumor cell self-protection. Mechanistically, KYNU and its metabolite 3-hydroxyanthranillic acid (3-HAA) upregulated the expression of SOD2, thereby decreasing mitochondrial reactive oxygen species (mtROS). KYNU inhibitors affected the spatial overflow of mtROS from mitochondria to the endoplasmic reticulum (ER). Endoplasmic reticulum oxidoreductin 1α (ERO1α) was sensitively affected by KYNU-induced changes in the redox environment, stimulating the PERK-eIF2α-ATF4 pathway of the UPR^ER^. This in turn promoted oxidative folding, reduced the level of misfolded protein (MFP), and maintained tumor survival and progression. Additionally, ATF4 acted as a transcription factor in the KYNU promoter region, amplifying KYNU tumorigenesis in a positive feedback manner.

**Conclusion:**

M2-secreted KYNU promotes the malignant behavior and stemness remodeling of EC via the SOD2-mtROS-ERO1α-UPR^ER^ axis and establishes a positive feedback loop. Thus, KYNU is a potential therapeutic target for EC treatment.

**Supplementary Information:**

The online version contains supplementary material available at 10.1186/s13046-025-03285-y.

## Background

Endometrial cancer (EC) is the fifth most common cause of cancer-related death among women globally [1], and its incidence is significantly increasing. Despite early detection and various treatments, such as hysterectomy and radiation therapy, which are curative for most patients, the prognosis for those with advanced disease remains poor [2]. The 5-year overall survival rates for patients with stage IV-A and IV-B EC are only 17% and 15%, respectively [3]. Therefore, improving the long-term prognosis of patients with EC is a daunting challenge, and understanding the mechanisms underlying EC progression is essential for developing potential therapeutic strategies.

The recruitment and polarization of tumor-associated macrophages (TAMs) in EC are regulated by the EC tumor microenvironment (TME), leading to the formation of a predominantly M2-like macrophage infiltrate. TAMs promote lymphovascular angiogenesis through cytokine secretion, facilitate the immune evasion of endometrial cancer cells (ECCs) by interacting with other immune cells, and contribute to the development of EC through the secretion of exosomes [4]. Endometrial cancer stem cells (ECSCs) are a subpopulation of highly tumorigenic cells known to express various surface markers, such as CD44, CD24, and CD133, suggesting enhanced tumor pathogenicity [5, 6]. The role of ECSCs in tumorigenesis and cancer progression has also been addressed in our previous studies [7, 8]. While LSECtin, a transmembrane protein highly expressed in TAMs, enhances the stemness of breast cancer cells [9], the relationship between TAMs and the stemness transformation of ECCs has not yet been investigated. To explore the factors that influence the development of tumor cells in a macrophage-influenced microenvironment, we performed gene sequencing of an in vitro macrophage‒ECC coculture system. We observed that the Kynureninase (KYNU) gene regulates stemness transformation and influences multiple ECC phenotypes.

KYNU is located on chromosome 2q22.2 and encodes the enzyme kynureninase. This enzyme catalyzes the tryptophan catabolism of L-kynurenine and L-3-hydroxykynurenine to anthranilic acid and 3-hydroxyanthranilic acid, respectively, and plays a key role in tryptophan synthesis of NAD cofactors. KYNU is associated with immunosuppression in various tumors and affects the overall survival of patients [10]. Bioinformatics data suggest that KYNU expression levels in the endometrium correlate with pregnancy status [11]; however, its role in EC has not been reported.

While investigating the tumor-promoting mechanism of KYNU, we found that superoxide dismutase-2 (SOD2) interacts with KYNU and is highly expressed in a coculture system. SOD2, a member of the metal peroxide dismutase family, is an important endogenous antioxidant enzyme [12]. Oxidative stress maintains the dynamic balance of cellular biological processes and is a double-edged sword for tumors and their microenvironments. Although reactive oxygen species (ROS) recruit neutrophils and macrophages to kill cancer cells, they also support cancer cell development through tumor-promoting immune cells [13]. Thus, oxidative stress, macrophages, and tumors are inextricably linked. The flavonoid oroxylin has been reported to inhibit macrophage sepsis via the ASIRT3-SOD2-ROS pathway, reducing endometrial inflammation and fibrosis and ultimately treating uterine adhesions [14]. SOD2 overexpression has been observed in EC using mRNA microarrays and RT‒qPCR [15]. Our study confirms that the TME promotes further secretion of KYNU by M2 macrophages, enhancing EC stemness transformation by regulating SOD2.

Endoplasmic reticulum oxidoreductin 1α (ERO1α; also known as ERO1A or ERO1L) is a flavin adenosine dinucleotide (FAD)-containing ER-resident thiol oxidoreductase that works together with protein disulfide isomerase (PDI) to catalyze disulfide bond formation in nascent polypeptides. ERO1α confers a faster growth rate and a more aggressive phenotype to tumor cells by promoting angiogenesis and differentiation of immunosuppressive cells and inducing an immunosuppressive TME, leading to immune cell dysfunction. These functions depend largely on the extent of oxidative protein folding [16], which is highly regulated in the endoplasmic reticulum (ER). In many tumors, such as breast cancer and lymphoma, disruption of ER homeostasis caused by oncogenic and metabolic abnormalities in the TME triggers a state of persistent ER stress (ERS). Aberrant activation of ERS sensors and the UPR^ER^ signaling pathway has emerged as a vital regulator of tumor growth and metastasis [17, 18]. The PERK-eIF2α pathway is an integral part of the ERS signaling pathway. Protein kinase RNA-like ER kinase (PERK) attenuates the ability of bone marrow-derived macrophages to polarize into M2 macrophages [19] and enhances immune evasion of tumors [20]. Our study confirms that ERO1α overexpression activates the PERK-eIF2α-ATF4 pathway and reduces the number of misfolded proteins (MFPs) in ECCs and ECSCs.

In conclusion, our study aimed to elucidate the mechanism by which M2 macrophage-secreted KYNU influences the malignant biological and stemness remodeling of EC via the SOD2-mtROS-ERO1α-UPR^ER^ pathway in a coculture system. Additionally, activating transcription factor-4 (ATF4) acted as a transcription factor for KYNU, forming a positive feedback loop in EC. Our study provides insights on the regulatory mechanisms of EC stemness, potentially offering new therapeutic targets.

## Methods

### Clinical specimens

Human EC surgical and normal samples were obtained from the Shengjing Hospital of China Medical University in Liaoning, China. Informed consent was obtained from all patients, and our study was approved by the hospital’s ethics committee. None of the patients experienced complications before surgery, nor had they received any antitumor therapies. After histopathological diagnosis, the specimens were categorized according to the 2013 International Federation of Gynecology and Obstetrics staging system. The fresh tissues were stored at −80 °C for further use, while the paraffin-embedded EC sections were stored at room temperature. All procedures complied with the principles of the Declaration of Helsinki.

### Reagents

Recombinant human KYNU (MCE, HY-P75903), the KYNU inhibitor 3-hydroxyhippuric acid (MCE, HY-113085), mito-TEMPO (MCE, HY-112879), and 3-hydroxyanthranillic acid (3-HAA) (Sigma, 148,776, 0.1 μM) were used in this study. Cell counting kit-8 (CCK-8) reagent was used to determine the half-maximal effective concentration (EC50) and half-maximal inhibitory concentration (IC50) values for recombinant KYNU and its inhibitor. Four concentrations were used: 0.5 ng/ml, 1 ng/ml, 2 ng/ml, and 4 ng/ml for the EC50 and 50 μmol/L, 100 μmol/L, 500 μmol/L, and 1,000 μmol/L for the IC50. Statistical analysis was conducted using GraphPad Prism.

### Cell lines and cell culture

The Ishikawa cell line was provided by the Department of Pathophysiology at Peking University, Beijing, China. These cells were cultured in RPMI 1640 medium (Pricella Biotechnology, PM150110, Wuhan, China) supplemented with 10% fetal bovine serum (FBS; Pricella Biotechnology, 164,210). Human embryonic kidney (HEK) 293 T cells were obtained from the Shanghai Institute of Cell Biology of the Chinese Academy of Sciences (Shanghai, China) and maintained in Dulbecco’s modified Eagle’s medium/high-glucose medium (iCell Bioscience, iCell-0001, Shanghai, China) supplemented with 10% FBS. The human THP-1 cell line was purchased from American Type Culture Collection (TIB202).

Macrophages were induced from logarithmic growth phase THP-1 cells by treatment with 100 ng/ml PMA (Absin, abs9107; Shanghai, China) for 48 h. M1 and M2 macrophages were further induced from these macrophages using LPS (100 ng/ml, Sigma, L2880, Saint Louis, USA), IFN-γ (20 ng/ml, PeproTech, 300–02, NJ, USA), IL-4 (20 ng/ml, PeproTech, 200–04), and IL-13 (20 ng/ml, PeproTech, 200–13) for 48 h. All the macrophages were maintained in RPMI 1640 medium supplemented with 10% FBS.

The Ishikawa cell line was used for isolating ECSCs, which were cultured in serum-free medium (SFM) containing DMEM/F12 (1:1) (Pricella Biotechnology), 2% B27 supplements (Gibco), 20 ng/ml epidermal growth factor (EGF, Proteintech, HZ-1326, Wuhan, China), basic fibroblast growth factor (bFGF, Proteintech, HZ-1285), HEPES (Amresco, Solon, USA), 1% insulin-transferrin-selenium, 5% bovine serum albumin (BSA) (Roche, Basel, Switzerland), and 1% penicillin‒streptomycin (Invitrogen). ECSCs were suspended in 6-well low-attachment surface plates (Corning, 3471, NY, USA). The cells were maintained at 37 °C in a humidified incubator with 5% CO_2_. A coculture system of M2 macrophages with ECCs/ECSCs was established in 6-well chambers (0.4 μM pore size, BIOFIL, Guangzhou, China). The cells were incubated under hypoxic conditions in a Tri-gas incubator (Likang, E5019, Shanghai, China) at 1% O_2_.

### Flow cytometry

To measure the ROS levels, the cells were resuspended in phosphate-buffered saline (PBS) and stained with 10 μM MitoSOX Red (MCE, HY-D1055, NJ, USA) for 15 min in the dark. Flow cytometry was used for analysis at an excitation wavelength of 510 nm and an emission wavelength of 580 nm. For cell surface marker analysis, the cells were stained with fluorescent-conjugated antibodies against APC-CD133 (BD Biosciences, 566,597, NJ, USA) and PE-CD44 (BD, 561,858) for 30 min. Blank and single-stained groups were established concurrently.

For apoptosis analysis, the Annexin V-fluorescein isothiocyanate/propidium iodide (FITC/PI) Apoptosis Detection Kit (APExBIO, K2003, Houston, USA) was used according to the manufacturer’s instructions. Briefly, 5 μl of FITC Annexin V and 5 μl of PI were added to the suspended cells for 15 min in the dark. All the samples were analyzed using a BD FACSCalibur flow cytometer (NJ, USA).

For stem cell sorting, trypsin–EDTA (0.25%, NCM, C100C1, Suzhou, China) was used to digest Ishikawa cells into single cells after they were suspended in stem cell culture medium for approximately 24 h. A single-cell suspension (100 μL buffer/10^7^ cells) was incubated with CD133 and CD44 antibodies at 4 °C. The CD44 + CD133 + cells sorted by flow cytometry were retained and cultured as ECSCs.

For M1 and M2 macrophage sorting, THP-1 cells were induced into M1 and M2 macrophages using the reagents mentioned above, and the macrophages were labeled with specific antibodies. An anti-CD68 antibody was used to label all the macrophages. CD86 + cells are referred to as M1 macrophages, whereas CD163 + and CD86 + /CD163 + cells are identified as M2 macrophages. The sorting function was used to collect M1 and M2 macrophages after setting appropriate gating conditions on the flow cytometer.

### Reverse transcription and quantitative real-time PCR (qRT‒PCR)

Total RNA was extracted from fresh tissues or cells using TRIzol reagent (Takara, T9108, Dalian, China). Complementary DNA (cDNA) was synthesized from total RNA using the Hiscript Il Q RT SuperMix for gPCR (+ gDNA wiper) kit (Vazyme R223, Nanjing, China). qRT‒PCR was performed on an ABI Prism 7500 Fast Real-Time PCR System (Applied Biosystems, StepOnePlus, USA) using ChamQ Universal SYBR qPCR Master Mix (Vazyme, Q711) with specific PCR primers (Sangon Biotech Co., Ltd., Shanghai, China). β-actin served as the normalization control. The fold changes were calculated using the 2-ΔΔCT method. The primer sequences are provided in Table S1.

### Western blot

Total protein was extracted from the cells using radioimmunoprecipitation assay buffer (Beyotime Biotechnology, P0013B, Shanghai, China) supplemented with 1 mM phenylmethanesulfonyl fluoride (Beyotime Biotechnology, ST506). Proteins were separated by 10% sodium dodecyl sulfate‒polyacrylamide gel electrophoresis (SDS‒PAGE) and then transferred to polyvinylidene difluoride membranes (Millipore, IPVH00010, Massachusetts, USA). The membranes were blocked with 5% BSA and incubated with diluted primary antibodies (Table S2) at 4 °C overnight. The corresponding secondary antibodies were incubated at room temperature for 2 h. The antigen‒antibody reactions were visualized using ECL western blot Substrate (Abbkine, BMU101-CN, Wuhan, China) and Image Lab software (Bio-Rad, CA, USA). The relative integrated density values (IDVs) of the protein bands were normalized to those of β-Tubulin or β-Actin and calculated using ImageJ software.

### Cell proliferation assay

Cell proliferation was assessed using a CCK-8 assay (Glpbio, GK10001, California, USA) according to the manufacturer’s protocol. Approximately 5 × 10^3^ cells were seeded in 96-well plates. After appropriate cell culture, 10 μl of CCK-8 reagent was added to each well. The cells were then incubated for 4 h at 37 °C. OD450 absorbance values were measured using a SpectraMax M5 microplate reader (Molecular Devices, USA) at daily intervals.

### Tumorsphere cultivation

ECSCs harvested from monolayer cells were seeded in ultralow attachment 6-well plates containing stem cell culture medium at a density of 1 × 10^4^ cells/ml. The cells were incubated at 37 °C with 5% CO_2_ for 7–10 days. For secondary tumorsphere formation, a 40 µm cell strainer (Falcon, 352,340, Corning Corporate, Corning, NY, USA) was used to collect primary tumorspheres. Single-cell suspensions were reseeded into ultralow attachment plates at a density of 5 × 10^3^ cells/ml after disaggregating with accutase (Invitrogen, 00–4555-56, Carlsbad, CA). The cells were then incubated for another 7–10 days under the same conditions. Tumorspheres with diameters greater than 150 μm were counted.

### Cell transfection

The overexpression plasmids (pcDNA3.1-SOD2 and pcDNA3.1-ERO1α) and their respective negative controls (NCs) were synthesized by Zebrafish Biotech (Nanjing, China). Lipo3000 transfection reagent (Glpbio, GK20006) was used for plasmid transfection according to the manufacturer’s instructions. HBLV-LUC-PURO was purchased from HANBIO (Shanghai, China). The transfection efficacy was confirmed via qRT‒PCR. The plasmid sequences are listed in Table S3.

### Transwell invasion assay

A total of 1 × 10^5^ cells were resuspended in 200 μl of serum-free medium and seeded into the upper chamber of 24-well transwell chambers (8 μM pore size, Corning, NY, USA) precoated with Matrigel solution (BD, NJ, USA). Then, 500 μl of complete medium was added to each lower chamber. The invading cells on the lower membrane surface were fixed with 4% paraformaldehyde and stained with 0.1% crystal violet after 24 h of incubation. Three random fields were counted, and the cell numbers were calculated using ImageJ software.

### Chromatin immunoprecipitation (ChIP)

Following the manufacturer's protocol, the ChIP assay was performed using a ChIP Assay Kit (Beyotime, P2078, Shanghai, China). Briefly, cells were preprocessed with 37% formaldehyde and lysed with SDS lysis buffer supplemented with 1 mM PMSF, followed by sonication to fragment the DNA into fragments between 200 and 1,000 bp for immunoprecipitation, and 1% agarose gel electrophoresis was used to detect the fragment length. The cell lysates were then incubated with antibodies (anti-ATF4 for the IP group, anti-RNA polymerase II antibody for the positive control group, and normal rabbit IgG for the negative control group) with protein A + G agarose.

at 4 °C overnight. The proteins and DNA complexes were eluted and de-cross-linked using 5 M NaCl.

at 65 °C for 4 h. The immunoprecipitated DNA was then purified with 0.5 M EDTA, 1 M Tris (pH 6.5) and 20 mg/ml proteinase K and detected via qRT‒PCR. The primers were designed on the basis of the predicted binding sites in the promoter region, and their sequences are listed in Table S1.

### Immunohistochemistry staining

Paraffin-embedded EC sections were obtained from the Pathology Department of the Shengjing Hospital, China Medical University. Immunohistochemistry (IHC) analysis was performed using an Immunohistochemical UltraSensitive™ SP Assay Kit (MXB, KIT-9710, Fuzhou, China). Each section was deparaffinized and rehydrated, followed by antigen retrieval in citrate buffer at boiling temperature for 10 min. After incubation with endogenous peroxidase, the sections were blocked with normal goat serum and incubated again with primary antibodies against CD206 (marker for M2 macrophages; 1:50; Proteintech) and HLA-DRα (marker for M1 macrophages; 1:1,000; Abcam, ab92511; Cambridge, UK) at 4 °C overnight. The bound primary antibodies were visualized using the DAB Horseradish Peroxidase Color Development Kit (MXB, DAB-0031). Morphological images were captured using a confocal laser microscope (Olympus Optical, Tokyo, Japan).

### Immunofluorescence assay

Immunofluorescence assays were performed using an Immunofluorescence Staining Kit (Elabscience, E-IR-R326/321, Wuhan, China). Paraffin-embedded sections were deparaffinized, rehydrated, and subjected to antigen retrieval. Both paraffin-embedded tissues and cells were fixed with 4% paraformaldehyde for 20 min, permeabilized with 0.2% Triton X-100 for 15 min, and blocked with normal goat serum for 30 min. Primary antibodies against HLA-DRα (1:300, Abcam), CD206 (1:50, Proteintech), KYNU (1:50, Thermo Fisher), and ERO1α (1:200, Proteintech) were incubated overnight at 4 °C. This was followed by incubation with fluorescent dye-labeled secondary antibodies at room temperature for 2 h. The nuclei were stained with DAPI. MFP detection was performed using the PROTEOSTAT® Aggresome Detection Kit (Enzo Life Sciences, ENZ-51035, NY, USA) following the manufacturer’s instructions. Briefly, the cells were fixed and permeabilized after incubation with MG132 for 18 h. Staining was performed via PROTEOSTAT and Hoechst. The ER was stained with ER-Tracker Blue‒White DPX (Glpbio, GB30170), the mitochondria were stained with MitoTracker Green (Beyotime, C1048), and MitoSOX Red (MCE, HY-D1055) was used to stain for mitochondrial reactive oxygen species (mtROS). Images were captured using a confocal laser microscope (Olympus, Tokyo, Japan). Superresolution imaging of the mitochondrial structures was performed using a highly intelligent and sensitive SIM system from Guangzhou CSR Biotech Co. Ltd., with images acquired using a 100 × /1.5 NA oil immersion objective (Olympus).

### Enzyme-linked immunosorbent assay (ELISA)

Macrophages and endometrial cell lines were cultured under standard conditions. The supernatants were collected after centrifugation. For the ELISA, 100 μL of the supernatant was added to each well of a 96-well plate precoated with 0.5 μg of KYNU antibody and incubated overnight at 4 °C. The plates were then blocked with 0.1% BSA solution for 1 h at 37 °C. KYNU expression levels in the supernatants were then detected using a SignalUp™ ELISA kit (Beyotime, P0205S) according to the manufacturer’s instructions. To quantify the KYNU levels, a SpectraMax M5 microplate reader (Molecular Devices, Sunnyvale, CA, USA) was used to read the OD450 values.

### Coimmunoprecipitation (Co-IP) assay

Coimmunoprecipitation (Co-IP) assays were performed using the Pierce Coimmunoprecipitation (Co-IP) Kit (Thermo, 26,149). Traditional co-IP methods that use proteins A or G result in the coelution of antibody heavy and light chains that may comigrate with relevant bands, masking important results. However, the Pierce Co-IP Kit covalently couples antibodies to an amine-reactive resin to avoid this issue. Briefly, 50 μg of antibodies against KYNU and SOD2 for the IP groups and normal IgG for the negative control group were immobilized on agarose resins for 2 h at room temperature. ECCs and ECSCs were collected, washed with PBS, and lysed in IP lysis buffer. The supernatants were then incubated with the prepared agarose resins overnight at 4 °C. The immunoprecipitated complexes were collected using agarose resin, centrifuged, washed, and analyzed by western blot.

### Luciferase assay

The predicted ATF4 binding sequence in the KYNU promoter region and its mutant sequences were subsequently cloned and inserted into pGL3 dual-luciferase vectors (Zebrafish Biotech). Transient transfection of the ATF4 plasmid and pGL3-KYNU-promoter (human, wt/mut)-luc + -SV40 into HEK293T cells was performed using Lipofectamine 3000 (Invitrogen) according to the manufacturer’s instructions. A dual-luciferase reporter assay system (Promega, Madison, WI, USA) was used to measure luciferase activity 48 h posttransfection. Renilla activity was used as an internal control.

### TCGA data processing

Gene expression correlation analyses of KYNU and SOD2 were conducted using data from The Cancer Genome Atlas (TCGA) (https:// cancergenome nih gov/). The dataset included a total of 589 patients with EC.

### Mouse xenografts

The animal experiments were approved by the Scientific Research and New Technology Ethics Committee of Shengjing Hospital of China Medical University (No. 2023PS408K). Five-week-old female athymic BALB/c nude mice were purchased from HFK Bioscience Co., Ltd. (Beijing, China) and were randomly assigned groups, with each mouse receiving a subcutaneous injection. Every week before injection, the cells were pretreated with a KYNU inhibitor for 48 h according to the manufacturer’s instructions or were pretransfected with a SOD2 overexpression plasmid. For the i-KYNU + SOD2-OE group, the transfected cells were pretreated with a KYNU inhibitor for 48 h before injection. A total of 1 × 10^3^ ECSCs or 1 × 10^7^ ECCs were collected and injected into the axilla of the nude mice. The tumor volume was measured every 4 days starting from Day 12 after injection using the following formula: $tumor volume (mm3) = \frac{{length \times width}^{2}}{2}$. Prior to sacrifice, the mice were anesthetized with isoflurane and imaged using D-luciferin (Glpbio, GC43496) and the Carestream Image Station System. The transplanted tumors were excised, weighed, and prepared for further analysis after the nude mice were sacrificed. The overall process of subcutaneous tumor transplantation in mice is displayed in Fig. S1H. For cell surface marker analysis, tumor tissues were minced and homogenized and then digested with a solution of collagenase I and deoxyribonuclease I in a cell culture incubator at 37 °C for 30 min. The cell suspension was obtained by filtering through a 70 μm cell filter for further analysis by flow cytometry. For the ROS staining assays, fresh tissues were embedded in OCT medium.

### Statistical analysis

All experimental data are presented as the means ± SDs from at least three independent experiments. The significance of different comparisons was analyzed using GraphPad Prism Software 9.0 (La Jolla, CA, USA). Comparisons between two groups were made using Student’s t test, whereas one-way analysis of variance (ANOVA) was used to compare multiple groups. Pearson ${x}^{2}$ tests were used to determine correlations. Statistical significance was set at *P* < 0.05.

## Results

### M2 macrophages promote the tumorigenic processes of EC

**Fig. 1:**
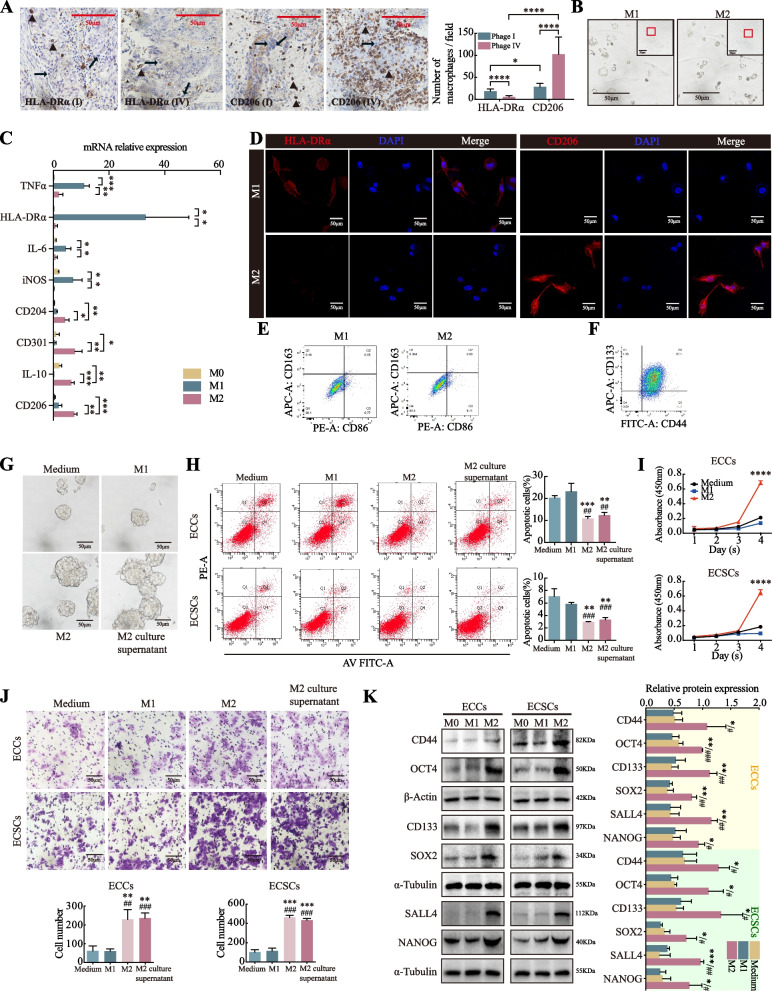
M2 macrophages promote the tumorigenic processes of EC. **A** M1 and M2 macrophage infiltration was detected by HLA-DRα and CD206 antibodies, respectively. The quantity of infiltrated M2 macrophages in stage IV (*n* = 17) EC was greater than that in stage I (*n* = 34) EC, as detected by immunohistochemical staining. **B** Monocytes were induced to M0, then M1 and M2 macrophages, and the cellular morphology is displayed. **C** Markers of M1 and M2 macrophages were measured by qRT‒PCR for induction identification (TNFα, HLA-DRα, IL-6, and iNOS for M1, CD204, CD301, IL-10, and CD206 for M2). **D** The classical biomarkers HLA-DRα and CD206 in M1 and M2 macrophages were detected via an immunofluorescence assay. **E** Induced M1 and M2 macrophages were sorted by flow cytometry. CD86 + cells are referred to as M1 macrophages, whereas CD163 + and CD86 + /CD163 + cells are referred to as M2 macrophages. **F** CD44 + /CD133 + ECSCs were sorted by flow cytometry. **G** Effects of M1 and M2 macrophage and M2-ECC coculture supernatants on the mammosphere formation of ECSCs in the coculture system. **H** Effects of M1 and M2 macrophage and M2-ECC coculture supernatants on the apoptosis of ECCs and ECSCs, as determined by flow cytometry. **I** Effects of M1 and M2 macrophages on the proliferation of ECCs and ECSCs, as determined by a CCK-8 assay. **J** Effects of M1 and M2 macrophage and M2-ECC coculture supernatants on the cell invasion of ECCs and ECSCs, as determined via a transwell assay. **K** Western blot analysis of the effects of M1 and M2 macrophages on the expression of tumor cell stemness biomarkers. The data are presented as the means ± SDs (*n* = 3 per group), Student’s t test, one-way analysis of variance (ANOVA). *P < 0.05, **P < 0.01, ***P < 0.001, ****P < 0.0001 for the M2 group/M2 culture supernatant group compared with the medium group. ##*P* < 0.01 and ###*P* < 0.001 for the M2 group/M2 culture supernatant group compared with the M1 group

To determine which types of macrophages are involved in the TME of EC tumorigenesis, immunohistochemical staining was performed. The results revealed greater infiltration of M2 macrophages than M1 macrophages in EC tissues. Moreover, the number of M2 macrophages was significantly greater in stage IV cancer tissues than in stage I EC tissues (Fig. 1A). These findings suggest that M2 macrophages are recruited by ECCs and are quantitatively related to cancer stage.

THP-1 monocytes were then induced to differentiate into M0 cells, followed by M1 and M2 macrophages (Fig. 1B). The expression levels of the associated marker genes were analyzed via qRT‒PCR (Fig. 1C). TNFα, HLA-DRα, IL-6, and iNOS for M1 macrophages were highly expressed in polarized M1 macrophages, whereas CD204, CD301, IL-10, and CD206 for M2 macrophages were highly expressed in polarized M2 macrophages. The results of the immunofluorescence assay of the macrophage markers were consistent with the qRT‒PCR results (Fig. 1D). The induced cells were sorted using flow cytometry with appropriate antibodies to achieve increased purity. An anti-CD68 antibody was used to sort all macrophages. CD86 + cells were identified as M1 macrophages, whereas CD163 + and CD86 + CD163 + cells were referred to as M2 macrophages (Fig. 1E, S1A). These results confirmed the successful polarization of M1 and M2 macrophages.

ECSCs were sorted using flow cytometry in a similar manner. CD133 + CD44 + cells were selected and further cultured (Fig. 1F). The role of M2 macrophages in tumorigenesis was assessed by coculture with ECC/ECSCs. M2 macrophages increased mammosphere formation in ECSCs (Fig. 1G). A flow cytometry assay revealed a lower apoptotic rate in the groups cocultured with M2 macrophages (Fig. 1H, S3C). Additionally, the proliferative and invasive capacities of cancer cells were significantly increased when they were cocultured with M2 macrophages (Fig. 1I, J, S3A, B). Notably, similar results were obtained when ECCs/ECSCs were cultured in the supernatant from the ECC/ECSC-M2 macrophage coculture system. In contrast, the proliferation, invasion, and apoptotic capacities of ECCs/ECSCs cocultured with M0 or M1 macrophages remained unchanged. M2 macrophages presented increased expression of stemness markers (CD44, OCT4, CD133, SOX2, SALL4, NANOG), whereas M0 and M1 macrophages did not (Fig. 1K). These results suggest that the supernatant from the M2 macrophage and ECC/ECSC coculture system played a crucial role in promoting tumorigenesis.

### KYNU promotes the malignant behavior of ECCs and ECSCs

**Fig. 2:**
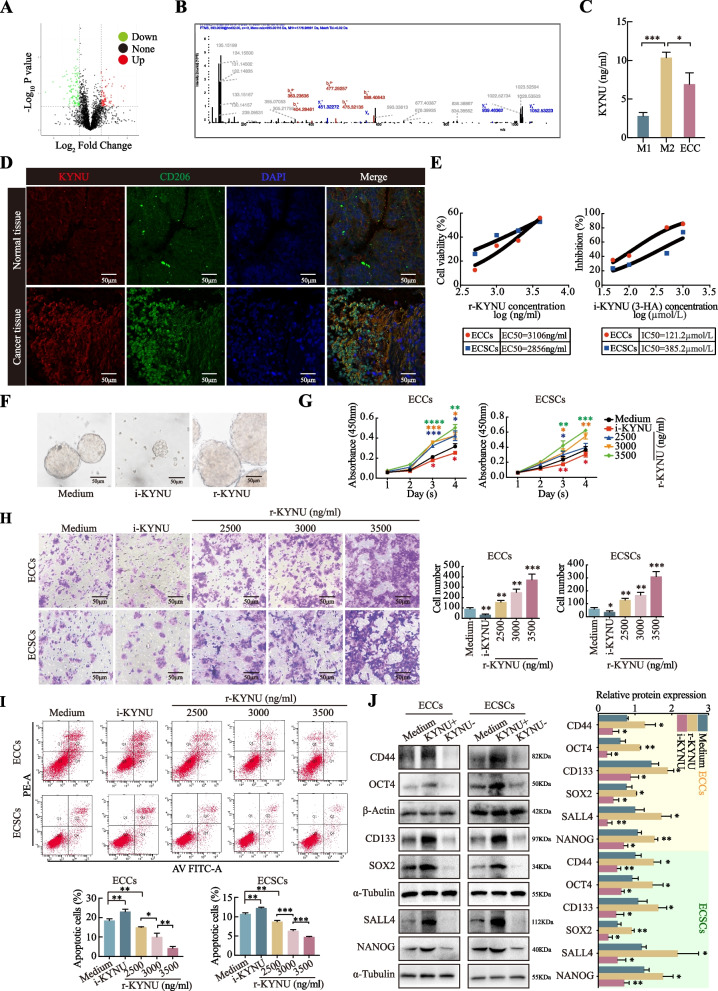
KYNU promotes the malignant behavior of ECCs and ECSCs. **A** Volcano diagram generated by TMT Quantitative Proteomics Technical Services revealed 2,914 proteins, among which 97 proteins were highly expressed and 106 proteins were expressed at low levels. **B** Best-matched peptide fragment spectrogram information of KYNU. **C** KYNU concentrations in the M1 macrophage, M2 macrophage and ECC supernatants determined via ELISA. **D** Double-staining immunohistochemistry analysis of KYNU and CD206 revealed higher expression levels in cancer tissues than in normal tissues. **E** The EC50 of recombinant KYNU and the IC50 of the KYNU inhibitor 3-HA were detected by a CCK-8 assay. Effects of r-KYNU and i-KYNU on mammosphere formation **F**, cell proliferation **G**, cell invasion **H**, and cell apoptosis **I** of ECSCs, and concentration gradients used. **J** Western blot analysis of the effects of r-KYNU and i-KYNU on the expression of tumor cell stemness biomarkers. The data are presented as the means ± SDs (*n* = 3 per group), Student’s t test, one-way analysis of variance (ANOVA). *P < 0.05, **P < 0.01, ***P < 0.001, ****P < 0.0001 compared with the medium group

To elucidate which protein contributed to the phenotypic changes in ECCs/ECSCs, we utilized TMT Quantitative Proteomics Technical Services from Novogene (Beijing, China). M2 macrophages, both routinely cultured and cocultured with ECCs for 48 h, were analyzed. The volcano diagram revealed 2,914 proteins, 97 of which were highly expressed and 106 of which were expressed at low levels (Fig. 2A). A corresponding heatmap is displayed below (Fig. S2). KYNU emerged as particularly notable because of its relatively high expression level, which was determined by the order of the fold change and p value. In total, eight peptide fragments of KYNU were identified in the proteomic assay, with the most compatible unique peptide fragment (Fig. 2B). The remaining seven peptide fragments are shown in Fig. S1B.

We then quantified KYNU expression in supernatants of the three types using ELISA. The results indicated that M2 macrophages secreted higher levels of KYNU than did M1 macrophages and ECCs, suggesting that M2 macrophages are the major source of KYNU in the TME (Fig. 2C). To map the KYNU protein distribution in cancer tissues, we conducted double-staining immunohistochemistry analysis using anti-KYNU and anti-CD206 antibodies. The analysis revealed that KYNU is predominantly localized in the stroma of EC tissues, with higher fluorescence intensities for both KYNU and CD206 in cancer tissues than in normal tissues (Fig. 2D). These findings suggest that KYNU plays a significant role in EC development.

Furthermore, we determined the optimal concentrations of recombinant (r-KYNU) and inhibitor (i-KYNU, 3-hydroxyhippuric acid [21]) KYNU by calculating the EC50 and IC50 values using the CCK-8 reagent. For Ishikawa cells, the EC50 of r-KYNU was 3,106 ng/ml, and the IC50 of i-KYNU was 121.2 μmol/L. For ECSCs, the values were 2,856 ng/ml and 385.2 μmol/L, respectively (Fig. 2E). For HEC-1A cells, the IC50 value was 193.7 μmol/L (Fig. S3D).

Our analysis revealed that the mammosphere formation ability of ECSCs was greater in the r-KYNU group than in the i-KYNU group (Fig. 2F). Further experiments with varying concentrations of recombinant KYNU demonstrated that increased r-KYNU levels enhanced cell proliferation and invasion, while the i-KYNU group showed the lowest levels of these activities (Fig. 2G, H). Additionally, higher concentrations of r-KYNU were associated with reduced apoptosis rates (Fig. 2I). We also examined the changes in the expression of six stemness markers, which increased with increasing r-KYNU and decreased with increasing i-KYNU (Fig. 2J). In summary, these data collectively suggest that KYNU promotes the malignant behavior of ECCs in a concentration-dependent manner.

### KYNU modulates the biological effects of cancer cells by regulating SOD2-mtROS

**Fig. 3:**
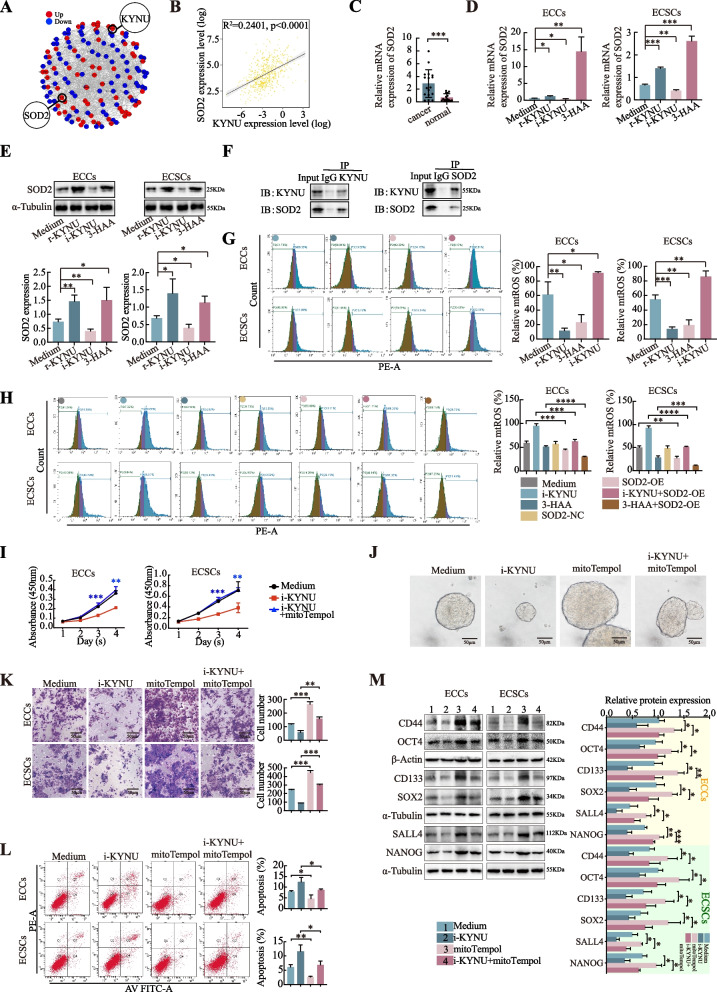
KYNU modulates the biological effects of cancer cells by regulating SOD2-mtROS. **A** Blast in the StringDB database based on the protein sequences of KYNU. Selected proteins, which were also differentiated and expressed in our TMT quantitative proteomics detection, are displayed. **B** Correlation between KYNU and SOD2 expression in 589 patients in the TCGA database according to Pearson's correlation analysis. **C** The expression level of SOD2 mRNA in EC tissues (*n* = 36) was greater than that in normal tissues (*n* = 110) according to qRT‒PCR. **D**, **E** KYNU and its metabolite 3-HAA increased the level of SOD2, whereas i-KYNU decreased it, as shown by qRT‒PCR and western blot. **F** Co-IP revealed the interaction between KYNU and SOD2 in ECCs. **G** KYNU and 3-HAA decreased the mtROS level, whereas i-KYNU increased it, as determined via flow cytometry. **H** SOD2 overexpression reduced the level of mtROS and abrogated the high level of mtROS induced by i-KYNU. Together, 3-HAA and SOD2 overexpression decreased mtROS to the greatest extent. Proliferation **I**, mammosphere formation **J**, cell invasion **K**, and cell apoptosis **L** of mitoTempol processed-treated cells and the rescue effect of mitoTempol on i-KYNU. **M** Western blot analysis of the effects of r-KYNU and mitoTempol on the expression of tumor cell stemness biomarkers. The data are presented as the means ± SDs (*n* = 3 per group), Student’s t test, one-way analysis of variance (ANOVA). *P < 0.05, **P < 0.01, ***P < 0.001, ****P < 0.0001

To investigate the mechanism by which KYNU influences cancer, we identified the sequences of proteins in the StringDB (V10) database and used these proteins to identify genes that may interact with KYNU. SOD2 emerged as a key candidate (Fig. 3A). We explored the significant correlation between KYNU and SOD2 expression using data from 589 patients in the TCGA database (Fig. 3B). Expression analysis revealed that SOD2 mRNA levels were significantly higher in fresh EC tissues (*n* = 110) than in normal tissues (*n* = 36) (Fig. 3C). The relationships between SOD2 expression and the clinical characteristics of EC patients are shown in Table S4, which shows that SOD2 expression is correlated with the stage and invasion depth of EC.

KYNU is a crucial enzyme in the tryptophan metabolic pathway. It catalyzes the conversion of 3-hydroxykynurenine (3-HK) to 3-hydroxyanthranillic acid (3-HAA), with NAD + being the main product of this process [22]. Adding r-KYNU or 3-HAA to the ECCs/ECSCs significantly increased the expression of SOD2, whereas i-KYNU decreased it (Fig. 3D). This trend was also consistent at the protein level, as confirmed by qRT‒PCR and western blot analysis (Fig. 3E, S3E). Co-IP assays further demonstrated interactions between KYNU and SOD2 (Fig. 3F, S3F). These results suggest that SOD2 is involved in the KYNU-mediated regulation of ECCs/ECSCs.

Since SOD2 catalyzes the reduction in mitochondrial reactive oxygen species (mtROS) and protects cells from oxidative stress damage [23], we further measured mtROS levels. The results revealed that mtROS levels were decreased by r-KYNU and 3-HAA and increased by i-KYNU (Fig. 3G). The transfection efficiency of the SOD2 overexpression plasmid was verified by qRT‒PCR and western blot (Fig. S1C). Cells overexpressing SOD2 exhibited low mtROS levels. Moreover, the high levels of mtROS attached to i-KYNU were abrogated by SOD2 overexpression, and mtROS levels were extremely low in combination with 3-HAA and SOD2 overexpression (Fig. 3H, S3G).

To further verify their inhibitory effect on mtROS, we incubated cells with i-KYNU or 3-HAA and mitoTempol, an mtROS-specific scavenger. MitoTempol reduces mtROS production. The high levels of mtROS induced by i-KYNU were abated by mitoTempol, with mitoTempol minimizing mtROS production together with 3-HAA (Fig. S1D). Cell proliferation (Fig. 3I, S3H), sphere formation (Fig. 3J), and cell invasion (Fig. 3K, S3I) assays revealed that mitoTempol abrogated the i-KYNU-induced inhibition of tumorigenesis and decreased the expression levels of stemness markers (Fig. 3M). The increased apoptotic rate was mitigated by mitoTempol treatment (Fig. 3L, S3J). In summary, KYNU upregulates SOD2 to reduce mtROS levels, thereby promoting the downstream biological effects of cell activation and stemness remodeling.

### ERO1α is responsible for the stemness-promoting and phenotype-changing effects of the KYNU-SOD2-mtROS axis

**Fig. 4:**
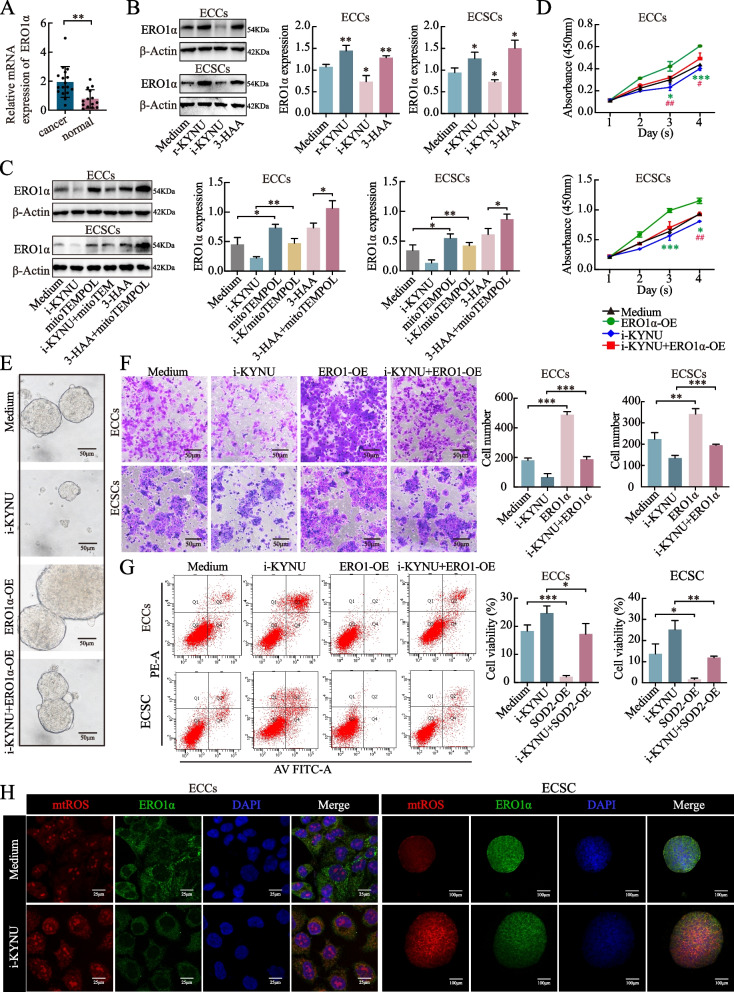
ERO1α participates in the stemness-promoting and phenotype-changing effects of the KYNU-SOD2-mtROS axis. **A** The expression level of ERO1α mRNA in cancer tissues (*n* = 110) was greater than that in normal tissues (*n* = 31). **B** KYNU and the metabolite 3-HAA increased the expression level of ERO1α, whereas i-KYNU decreased ERO1α expression, as shown by western blot. **C** The MtROS inhibitor mitoTempol upregulated the expression of ERO1α and rescued the inhibitory effect of i-KYNU. The KYNU metabolites 3-HAA and mitoTempol significantly maximized the promoting effect of ERO1α. The effect of ERO1α overexpression on cell proliferation **D**, mammosphere formation **E**, cell invasion **F**, and cell apoptosis **G**, and the rescuing effect of ERO1α on i-KYNU. **H** A KYNU inhibitor increased the level of mtROS and decreased the level of ERO1α. The subcellular locations of mtORS and ERO1α overlapped. The data are presented as the means ± SDs (*n* = 3 per group), Student’s t test, one-way analysis of variance (ANOVA). *P < 0.05, **P < 0.01, ***P < 0.001, ****P < 0.0001 compared with the medium group. #*P* < 0.05, ##*P* < 0.01 compared with the i-KYNU group

To further explore the potential mechanism by which the KYNU-SOD2-mtROS axis regulates stemness shaping and malignant behavior, we investigated the crucial oxidoreductase responsible for protein folding influenced by the REDOX state of TAMs. ERO1α was more highly expressed in EC tissues (*n* = 110) than in normal tissues (*n* = 31), as shown below (Fig. 4A). The relationships between ERO1α expression and the clinical characteristics of patients with EC are shown in Table S5. ERO1α expression was correlated with stage, lymphovascular space invasion, lymphatic metastasis and distal metastasis of EC. The protein expression levels of ERO1α decreased following treatment with i-KYNU and increased after r-KYNU or 3-HAA was added to the ECCs/ECSCs (Fig. 4B). Notably, mitoTempol upregulated ERO1α expression, counteracting the inhibitory effect of i-KYNU and significantly enhancing the promoting effect of 3-HAA (Fig. 4C, S3K). Our results indicate that ERO1α is crucial for the phenotypic remodeling of both ECCs and ECSCs. ERO1α overexpression restored the cell proliferation (Fig. 4D, S3L), sphere formation (Fig. 4E), and invasion (Fig. 4F, S3M) reduced by i-KYNU, and therefore decreased the percentage of apoptotic cells (Fig. 4G, S3N).

Mitochondria produce ROS as byproducts, which act as signaling molecules that diffuse into the cytoplasm to transmit stress signals. This signaling impacts the ER [24], and the UPR^ER^ process can be used to eliminate proteins that cannot fold properly into secondary structures. The mechanism of human ERO1α regulation is very delicate and is coordinated by the negative feedback of the disulfide bond on its flexible loop in the UPR^ER^. If the environment of the ER favors reduction, PDI in the reduced state opens the regulatory disulfide bond of ERO1α and activates it to promote oxidative folding. However, if the environment of the ER favors oxidation, PDI in the oxidized state shuts down the regulatory disulfide bond of ERO1α and inactivates it to prevent oxidative stress [25]. We found that KYNU inhibition increased mtROS levels and decreased ERO1α levels, with overlapping subcellular locations (Fig. 4H). These results suggest that ERO1α plays a vital role in the stemness-promoting process of the KYNU-SOD2-mtROS axis, potentially through mtROS.

### KYNU redistributes mtROS in the ER and activates PERK-eIF2α-ATF4 of the UPR^ER^ signaling pathway via ERO1α

**Fig. 5:**
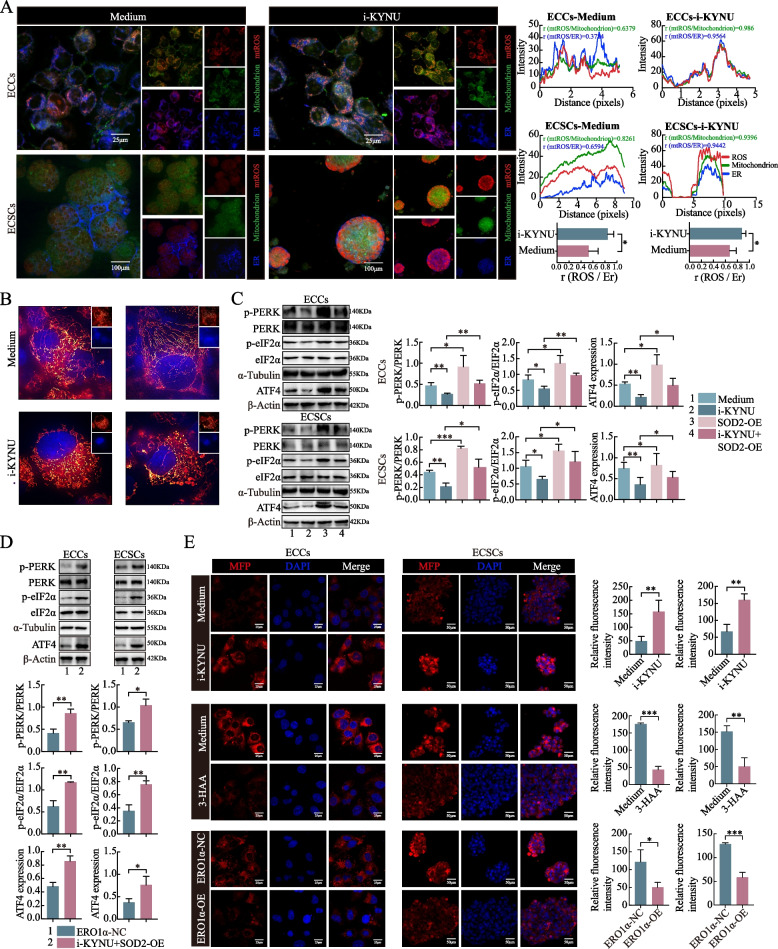
KYNU redistributes mtROS in the ER and activates PERK-eIF2α-ATF4 of the UPR^ER^ signaling pathway via ERO1α. **A** MtROS accumulated from the mitochondria to the ER under the influence of i-KYNU. Colocalization between the ER and mtROS was statistically significant. **B** The KYNU inhibitor caused the mitochondrial cristae to shorten or even break off, resulting in swelling within the cristae, widening of the crista gap, and the formation of vacuoles. **C** A KYNU inhibitor inhibited the phosphorylation of PERK and eIF2α but not their nonphosphorylated forms. SOD2 overexpression had the opposite effect and abolished the inhibitory influence induced by i-KYNU. **D** ERO1α overexpression upregulated p-PERK, p-eIF2α, and ATF4, and the original forms of PERK and eIF2α remained stable. **E** The KYNU inhibitor significantly increased the level of MFP in both ECCs and ECSCs, whereas 3-HAA and ERO1α overexpression decreased it. The data are presented as the means ± SDs (*n* = 3 per group), Student’s t test, one-way analysis of variance (ANOVA). *P < 0.05, **P < 0.01, ***P < 0.001 compared with the medium/i-KYNU/ERO1α-NC group

To determine the influence of mtROS on the ER, we examined the location of mtROS and found that it was located primarily in the mitochondria. However, when i-KYNU was added to the cells, mtROS accumulated in the ER. Quantitative statistical analysis of the colocalization data revealed a close spatial relationship between mtROS and the ER in the i-KYNU group (Fig. 5A). This finding was consistent with our hypothesis that the excess mtROS induced by i-KYNU were transported from the mitochondria to the ER. Superresolution imaging of mitochondrial structures was performed via commercial HIS-SIM. We observed that i-KYNU altered the mitochondrial structure, causing cristae shortening, swelling, gap widening, and vacuole formation (Fig. 5B). Compelling evidence has emerged that three signaling pathways are involved in the UPR^ER^: inositol-requiring enzyme-1, PERK, and activating transcription factor 6 signal transduction [26]. PERK is a cytoplasmic protein kinase that phosphorylates eIF2α under stress, inhibits its translation and increases the expression of transcription factors such as ATF4, which are involved in protein synthesis, folding, and the oxidative stress response. Phosphorylated eIF2α can also promote the expression of ATF4 [27]. We explored the protein expression levels of PERK, eIF2α, their activated phosphorylated forms, and ATF4. The results revealed that i-KYNU inhibited the phosphorylated forms of PERK and eIF2α but not their nonphosphorylated forms. However, as predicted, SOD2 overexpression had the opposite effect, abolishing the inhibitory effect induced by i-KYNU (Fig. 5C). We detected ERO1α overexpression at the mRNA and protein levels (Fig. S1E). We also observed that p-PERK, p-eIF2α, and ATF4 were upregulated by ERO1α overexpression, while the original forms of PERK and eIF2α remained stable (Fig. 5D). Additionally, the MFPs were significantly increased in the i-KYNU group but decreased in the 3-HAA and ERO1α overexpression groups (Fig. 5E). Our findings revealed that KYNU reduces mtROS redistribution from mitochondria to the ER, promoting a reductive ER environment via the KYNU-SOD2 axis. This environment upregulates ERO1α, activating PERK-eIF2α-ATF4 of the UPR^ER^ to eliminate the MFP for self-protection.

### ATF4 acts as a transcription factor of KYNU and contributes to a positive feedback loop

**Fig. 6:**
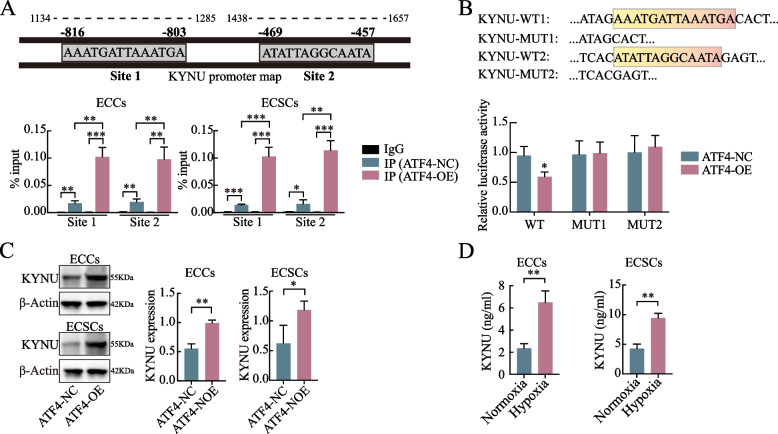
ATF4 acts as a transcription factor of KYNU and contributes to a positive feedback loop. **A** Two predicted binding sites of the KYNU promoter region with the transcription factor ATF4 were verified by a ChIP assay in both ECCs and ECSCs. **B** Two predicted binding sites of the KYNU promoter region with the transcription factor ATF4 were further validated via a dual-luciferase reporter assay. The fluorescence value was significantly lower in the ATF4-OE/KYNU-WT group than in the ATF4-NC or KYNU-Mut groups. **C** ATF4 overexpression increased the level of KYNU in ECCs and ECSCs, as shown by western blot. **D** ECCs/ECSCs cultured in a hypoxic environment presented increased KYNU levels in the medium. The data are presented as the means ± SDs (*n* = 3 per group), Student’s t test, one-way analysis of variance (ANOVA). *P < 0.05, **P < 0.01, ***P < 0.001 compared with the IgG/IP (ATF4-NC)/KYNU-WY + ATF4-OE/ATF4-NC/Normoxia group

ATF4 belongs to the ATF/CREB transcription factor family of alkaline leucine zipper domain proteins. They are highly expressed in many tumor cells and are associated with increased cell survival and stress resistance [28]. These tumor-supportive effects of ATF4 have been shown to be mediated by cancer-associated fibroblasts [29]. Using the JASPAR database (http://jaspar.Genereg.Net/cgi-bin/jaspar_db. Pl), we predicted that ATF4 has multiple binding sites in the KYNU promoter region. Thus, to further investigate whether ATF4 promoted KYNU tumorigenesis, we chose two binding sites with relatively high scores. Ultrasonic fragmentation was assessed using agarose gel electrophoresis, and the motif is shown below (Fig. S1F). The ChIP results revealed that ATF4 binds to two predicted sites in the promoter region of KYNU (Fig. 6A, S3O). We confirmed the interoperability using a dual-luciferase reporter assay. The cells were then cotransfected with the wild-type dual-luciferase vector containing the binding sequence of the KYNU promoter region and the ATF4 plasmid. We then observed that the fluorescence value was significantly lower than that of the ATF4-NC or mutant-type groups (Fig. 6B). Next, the cells were transfected with the ATF4 overexpression plasmid, and the transfection capacity was confirmed by qRT‒PCR and western blot (Fig. S1G). Notably, ATF4 overexpression upregulated the levels of KYNU in ECCs/ECSCs, indicating that ATF4 affects KYNU production by tumor cells (Fig. 6C). Furthermore, ECCs/ECSCs incubated in a hypoxic environment presented higher KYNU levels in their supernatants (Fig. 6D). Changes in the state of the TAM affected the levels of KYNU in M2 macrophages and tumor cells. These results suggest that ATF4 acts as a transcription factor for KYNU and promotes a cascade reaction through a positive feedback loop. This “self-strengthening” phenomenon coincides well with the finding that a hypoxic TAM synergizes with the effect of KYNU.

### A KYNU inhibitor hinders stemness and tumorigenesis via the SOD2-mtROS-ERO1α-UPRER^ER^ pathway in vivo

**Fig. 7:**
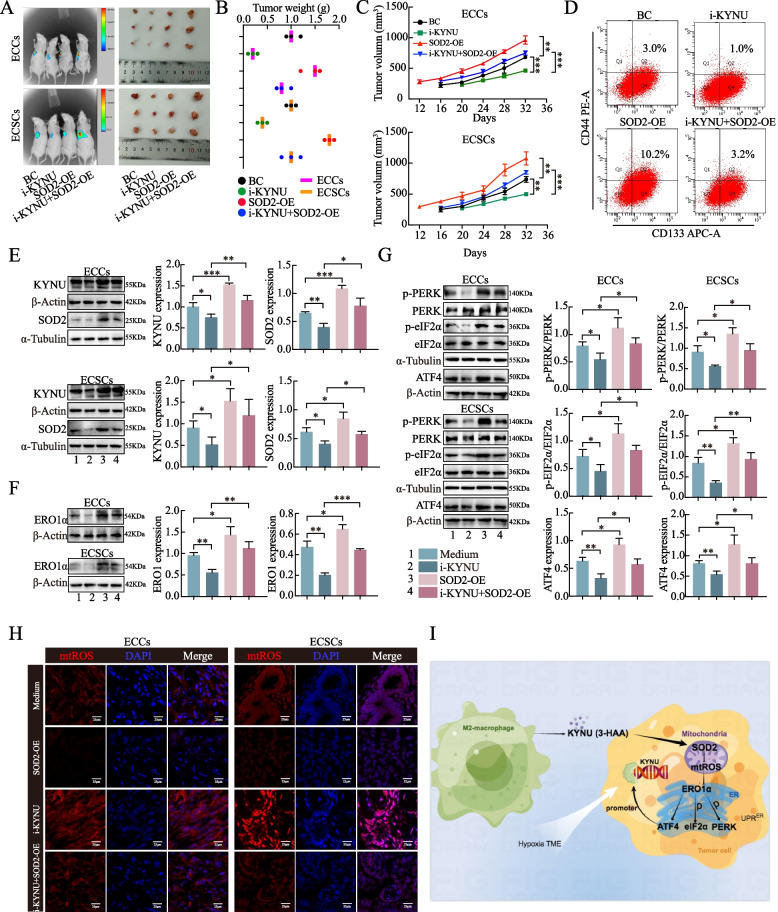
A KYNU inhibitor hindered stemness and tumorigenesis via the SOD2-mtROS-ERO1α-UPR^ER^ pathway in vivo. **A** Tumor-bearing nude mice and one tumor sample from each group are shown (*n* = 3). **B** Weights of each group of tumors after removal from the body. **C** Volume of each group of tumors at different observation times. **D** Proportion of CD44 + CD133 + cells in the i-KYNU- or SOD2-OE-xenografted tumors was detected by flow cytometry. **E** The expression of KYNU and SOD2 in i-KYNU- or SOD2-OE xenograft tumors was detected by western blot. **F** ERO1α expression in i-KYNU- or SOD2-OE-xenografted tumors was detected by western blot. **G** The expression of PERK/p-PERK, eIF2α/p-eIF2α, and ATF4 in the UPR^ER^ pathway in i-KYNU or SOD2-OE xenograft tumors was detected by western blot. **H** mtROS levels in i-KYNU- or SOD2-OE-xenografted tumors were detected by mtROS staining. **I** Schematic diagram of the KYNU-SOD2-mtROS-ERO1α-UPR.^ER^ axis mechanism. The data are presented as the means ± SDs (*n* = 3 per group), one-way ANOVA was performed. *P < 0.05, **P < 0.01, ***P < 0.001

The malignant effects of KYNU were next investigated in vivo. Overall, the weight and volume of the xenograft tumors in the i-KYNU group were lower than those in the SOD2 overexpression group. As anticipated, SOD2 overexpression mitigated the inhibitory effect of i-KYNU on tumors (Fig. 7A-C). The percentage of CD44 + CD133 + cells detected in the separated tumors of the ECC xenograft models was lowest in the i-KYNU tissues, whereas SOD2 overexpression mitigated the inhibition of these tumors (Fig. 7D). To confirm the results in the xenograft tumor models, the expression levels of KYNU and SOD2 in the tissues were verified using western blot (Fig. 7E). Furthermore, we found that the expression levels of ERO1α and UPR^ER^-related proteins were also downregulated by i-KYNU, but this effect was mitigated by SOD2 overexpression (Fig. 7F, G). mtROS staining revealed that mtROS levels were highest in i-KYNU-xenografted tumors, but SOD2 overexpression attenuated mtROS levels (Fig. 7H). These data indicate that KYNU inhibitors impair stemness and tumorigenesis via the SOD2-mtROS-UPR^ER^ pathway in vivo.

## Discussion

In recent years, molecular subgroups have been shown to have stronger prognostic potential than histopathological tumor characteristics [30]. This observation has opened new avenues in molecular classification-based diagnostics and new approaches to targeted therapy that complement traditional diagnosis and treatment methods.

Macrophages are a highly heterogeneous and plastic class of intrinsic immune cells that are widely distributed in major body tissues [31]. Under the influence of the local microenvironment, macrophages can polarize into different phenotypic subcell types and perform different functions [32, 33]. On the basis of their activation status, function, and secretion of cytokines, macrophages can be classified into classically activated M1 (proinflammatory) and selectively activated M2 (anti-inflammatory) macrophages [34]. EC is a hormone- and metabolism-dependent cancer; thus, TAMs may contribute to its development. This occurs when TAM interacts with metabolic factors such as the estrogen receptor and metabolic factors involved in glucose, lipid, and amino acid metabolism [35]. Chronic inflammation has been shown to be an important factor in cancer development. When TAMs are recruited to inflamed tissues, they release additional proinflammatory factors, such as IL-6, TNF, IFN-γ, EGF, various proteases, and ROS [36]. Together, these factors create a microenvironment that promotes the generation of mutations and enhances further proliferation of cancer cells. Different macrophage phenotypes are involved in different functions, and the interplay of these phenotypic variants determines the fate of tissues/organs during inflammation and injury. Previous studies have shown that M2 macrophages are highly tropic to hypoxic tumor regions. In these regions, the IRE1–XBP1 pathway, which is associated with ERS, is activated. This activation inhibits cytosolic glycolysis, promotes oxidative phosphorylation, and facilitates intracellular lipid accumulation, leading to the typical phenotype of M2-TAMs [37]. These findings suggest that manipulation of the ERS response of M2-TAMs may be a breakthrough in cancer therapy. To study tumor cell‒macrophage interactions accurately, we simulated the microenvironment in which tumor cells and macrophages coexist in vitro rather than focusing on one type of cell. Our study revealed that KYNU, which is secreted predominantly by M2 macrophages in EC tissues, regulates SOD2 in tumor cells. This alters mtROS levels, creating an altered environment that activates ERO1α in large quantities. This promotes the repair of proteins in the ER and reduces the amount of MFP available for cellular self-protection. KYNU initiates the ERS and UPR^ER^ pathways, leading to stemness remodeling of tumor cells, enhanced cell proliferation and invasion, and decreased apoptosis. Indeed, our TMT quantitative proteomics results revealed that many other proteins, such as apoptosis-associated speck-like protein containing CARD (PYCARD), DNA replication complex GINS protein PSF3 (GINS3), carbonic anhydrase 2 (CA2), lactotransferrin (LTF), ATP-binding cassette subfamily F member 3 (ABCF3), and pyruvate kinase (PKM2) were differentially expressed. PYCARD plays a crucial role in cell apoptosis and the inflammatory response [38], whereas GINS3 is a member of the GINS complex and plays an important role in initiating and prolonging chromosome replication since incorrect DNA replication events can lead to disease [39]. CA2, LTF, ABCF3, and PKM2 are involved in various energy and trace element metabolism processes in living organisms [40–43]. Therefore, the role of these upregulated proteins is consistent with the cancerous nature and metabolic abnormalities of endometrial cancer. In the general environment of endometrial cancer with M2 macrophage infiltration, numerous differentially expressed proteins work together to promote cancer occurrence and development.

KYNU is an intermediate enzyme in tryptophan metabolism, and evidence suggests that plays an important role in cancer. Tryptophan metabolism promotes tumor progression by suppressing antitumor immune responses and increasing the malignant properties of cancer cells [44, 45]. Apart from the well-known indoleamine 2,3-dioxygenase-1 and tryptophan 2,3-dioxygenase [46], studies on the mechanisms by which other key intermediates of tryptophan metabolism are involved in carcinogenesis are relatively rare. There is an even greater lack of studies on EC. Macrophages that overexpress KYNU promote the progression of gastric cancer through unique ligand‒receptor pairs and transcription factors. KYNU overexpression is also correlated with adverse clinical phenotypes of gastric cancers [47]. KYNU confers tumorigenic activity to gastric cancer cells through the production of 3-HAA, conferring anti-iron death properties [44]. Reduced systemic tryptophan levels have been observed in patients with various cancers, including adult T-cell leukemia, colorectal cancer, gynecological cancers, malignant melanoma, lung cancer, and malignant gliomas. However, elevated concentrations of kynurenine (Kyn) pathway metabolites have rarely been observed [48]. This may indicate more restricted local variation in Kyn and other downstream metabolites in the TME. Therefore, we hypothesized that intermediary metabolites may be involved in chemotaxis to metabolically advanced tumors and thus mediate tumor progression. Our study demonstrated for the first time that KYNU and its direct metabolite, 3-HAA, can activate the UPR^ER^ pathway by altering mtROS homeostasis between the mitochondria and the ER. This alteration is involved in various malignant biological behaviors and stemness remodeling of EC in a concentration-dependent manner. Investigating tumor development from a metabolic perspective indicates that KYNU and its metabolites may be potential new targets for EC therapy.

Oxidative stress acts as a double-edged sword, affecting the tumor environment in contradictory ways [49]. The same levels of ROS may have opposite effects on different tumors, and different levels of ROS may have varying impacts on the same tumor [50]. These differences arise from the nature of the ROS themselves or the varied responses of different tissues to ROS, allowing certain cells to selectively mitigate cell death while retaining their signaling capacity [51, 52]. However, the exact mechanisms of ROS remain to be fully investigated.

Our study focused on the dynamics of mtROS and revealed that under the influence of KYNU inhibitors, not only did the number of mtROS increase, but their spatial distribution also expanded from the mitochondria to the ER, affecting the ER redox environment. The main pathway for the oxidative folding of proteins involves ERO1α, a sulfhydryl oxidase, and PDI, a protein disulfide isomerase. PDI catalyzes the formation of disulfide bonds in substrate proteins, whereas ERO1α provides the upstream oxidative force to PDI [53]. ERO1α is highly expressed in a variety of tumors and is correlated with poor prognosis [54]. We found that the regulation of ERO1α was sensitive to KYNU-induced changes in the redox environment, which stimulate the PERK-eIF2α-ATF4 pathway, promote oxidative folding, reduce the number of MFPs, and thereby maintain tumor survival and progression.

Genomic instability, redox imbalance, a low-oxygen environment, nutritional deprivation, and other environmental stresses in tumor cells lead to the accumulation of MFPs in the ER, which produces toxic effects. Tumor cells maintain cellular proteostasis by activating the UPR^ER^ to increase MFP clearance [55, 56]. Phosphorylated PERK can activate the ERO1α‒CHOP pathway to mediate cellular biological behaviors [57], and ERO1α can form a complex with PERK through covalent interactions with the C-terminal active site and the cysteine 216 site of PERK [58]. These findings suggest a complex interaction between ERO1α and PERK, which warrants further studies for confirmation. Our study demonstrated that ERO1α regulates PERK, leading to its phosphorylation, which in turn activates eIF2α and ATF4 to adapt to ERS. Simultaneously, we observed that the amount of MFP in cells increased with KYNU inhibition, while its metabolite 3-HAA eliminated its accumulation. Interestingly, we determined that the transcription factor ATF4, which is highly expressed in various tumors, acts inversely on the promoter region of KYNU in tumor cells, resulting in an excess of KYNU in the TME. Moreover, the hypoxic environment of tumor cells stimulates KYNU secretion, suggesting a sophisticated cascade-amplified method of self-protection in response to external stress.

This study is the first to elucidate the role of KYNU and its metabolite 3-HAA in activating the UPR^ER^ pathway, providing a novel understanding of their impact on mtROS homeostasis and EC progression. Additionally, the use of various techniques, such as flow cytometry, fluorescence experiments, Co-IP, ChIP, and dual luciferase assays, ensured robust and detailed mechanistic insights. Furthermore, identifying KYNU as a potential therapeutic target opens new avenues for EC treatment strategies, offering significant clinical implications. While this study provides extensive in vitro data, in vivo validation is limited, which may affect the translatability of the findings to clinical settings. In addition, the focus on KYNU and its direct pathway may not account for other critical pathways and interactions in the tumor microenvironment that contribute to EC progression.

Finally, the relatively small sample sizes used in certain assays may limit the generalizability of the results across diverse patient populations.

## Conclusions

In conclusion, we elucidated that M2-secreted KYNU promotes the malignant behavior and remodeled stemness of EC via the SOD2-mtROS-ERO1α-UPR^ER^ axis, establishing a positive feedback loop. A schematic representation of this mechanism is shown in Fig. 7I. Thus, KYNU represents a potential therapeutic target for EC treatment.

## Supplementary Information

Supplementary Material 1: Fig S1. A Flow cytometry results of macrophage sorting. CD86+ cells are identified as M1 macrophages, whereas CD163+ and CD86+CD163+ cells are referred to as M2 macrophages. B Seven peptide fragments of KYNU from the proteomic assay are shown. C The transfection efficiency of the SOD2 overexpression plasmid was verified by qRT‒PCR and western blot. D MitoTempol rescued the high level of mtROS caused by i-KYNU. Together with 3-HAA, mitoTempol mostly decreased the level of mtROS. E The transfection efficiency of the ERO1α overexpression plasmid was verified by qRT‒PCR and western blot. F Ultrasonic fragmentation in the ChIP assay was detected using agarose gel electrophoresis, and the motif logo is shown. G The transfection efficiency of the ATF4 overexpression plasmid was verified via qRT‒PCR and western blot. H Diagram showing the time of tumor formation in BALB/c (nu/nu) mice transplanted with control or treated cells. The data are presented as the means ± SDs (*n* = 3 per group), Student’s t test, one-way analysis of variance (ANOVA). *P< 0.05, **P < 0.01, ***P < 0.001

Supplementary Material 2: Fig S2. The heatmap of the TMT Quantitative Proteomics Technical Services results. A total of 2,914 proteins were identified, with 97 highly expressed proteins and 106 proteins expressed at low levels.

Supplementary Material 3: Fig S3. Cell proliferative capacity A, cell invasion B, and cell apoptosis rate C of HEC-1A cells cocultured with M1 or M2 macrophages were detected by a CCK-8 assay, a transwell assay and flow cytometry. D IC50 values for the KYNU inhibitor in HEC-1A cells were detected by a CCK-8 assay. The concentrations used were 50 μmol/L, 100 μmol/L, 500 μmol/L, and 1,000 μmol/L. E The interaction between KYNU and SOD2 was verified by Co-IP. F The regulatory effects of the KYNU inhibitor, recombinant KYNU and 3-HAA on SOD2 were confirmed by western blot. G Flow cytometry analysis of the influence of KYNU, SOD2, MitoTempol and their combination on mtROS levels. The proliferative capacity H, invasion I, and apoptosis rate J of HEC-1A cells pretreated with the KYNU inhibitor or mitoTempol were detected by a CCK-8 assay, a transwell assay and flow cytometry. K western blot analysis of the influence of the KYNU inhibitors 3-HAA and mitoTempol as well as their combination on ERO1α expression. The cell proliferative capacity L, invasion M, and apoptosis rate N of the HEC-1A cells pretreated with the KYNU inhibitor or ERO1α-OE were detected via the CCK-8 assay, transwell assay and flow cytometry. O Two predicted binding sites of the KYNU promoter region with the transcription factor ATF4 were verified by a ChIP assay in the HEC-1A cell line. The data are presented as the means ± SDs (*n* = 3 per group), Student’s t test, one-way analysis of variance (ANOVA). *P < 0.05, **P < 0.01, ***P < 0.001.

Supplementary Material 4: Table S1. The sequences of primers used for qRT‒PCR and the binding sites in the promoter region of KYNU. Table S2. Information of the primary antibodies used in this study. Table S3. The plasmid sequences used in cell transfection. Table S4. Relationship of SOD2 expression with the clinical characteristics of patients with endometrial cancer. Pearson tests were used for statistical analysis. Table S5. Relationship of ERO1α expression with the clinical characteristics of patients with endometrial cancer. Pearson tests were used for statistical analysis.

## Data Availability

All data generated or analyzed during this study are included in this published article and its supplementary information files.

## Abbreviations

UPR^ER^: Endoplasmic reticulum unfolded protein response
3-HHA: 3-Hydroxyhippuric acid
3-HK: 3-Hydroxykynurenine
ATF4: Activating transcription factor-4
bFGF: Basic fibroblast growth factor
BSA: Bovine serum albumin
CCK-8: Cell counting kit-8
ChIP: Chromatin immunoprecipitation
Co-IP: Co-immunoprecipitation
EC: Endometrial cancer
EC50: Half-maximal effective concentration
ECC: Endometrial cancer cell
ECSC: Endometrial cancer stem cell
EGF: Epidermal growth factor
ELISA: Enzyme-linked immunosorbent assay
ER: Endoplasmic reticulum
ERO1α: Endoplasmic reticulum oxidoreductin 1α
ERS: Endoplasmic reticulum stress
IC50: And half-maximal inhibitory concentration (IC50)
i-KYNU: Inhibitor KYNU
KYNU: Kynureninase
MFP: Misfolded protein
mtROS: Mitochondrial reactive oxygen species
PBS: Phosphate-buffered saline
PDI: Protein disulfide isomerase
PERK: Protein kinase RNA-like ER kinase
r-KYNU: Recombinant KYNU
ROS: Reactive oxygen species
SOD2: Superoxide dismutase-2
TAM: Tumor-associated macrophage
TCGA: The Cancer Genome Atlas
TME: Tumor microenvironment
