# Supplementary figures and images for: M2 macrophage-secreted KYNU promotes stemness remodeling and malignant behavior in endometrial cancer via the SOD2-mtROS-ERO1α-UPRER axis
Source: J Exp Clin Cancer Res. 2025 Jul 4;44:193. doi: 10.1186/s13046-025-03285-y (PMC12231660; doi:10.1186/s13046-025-03285-y)

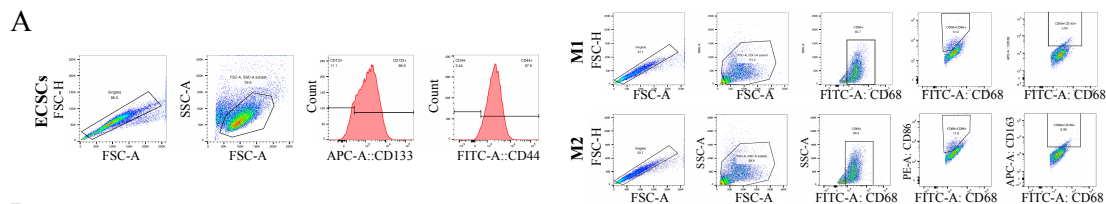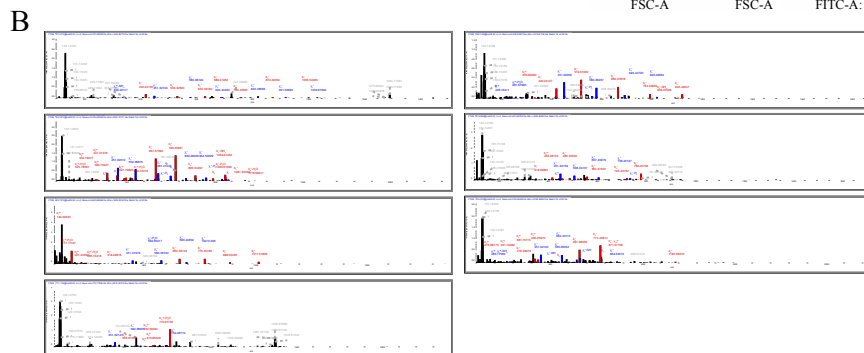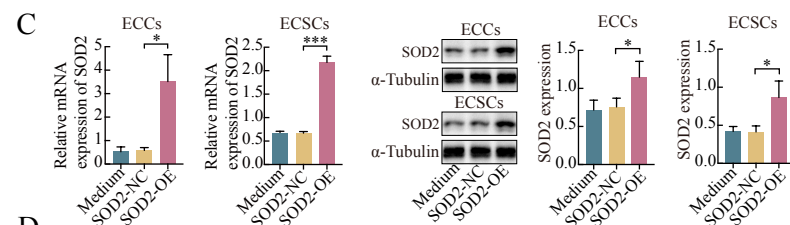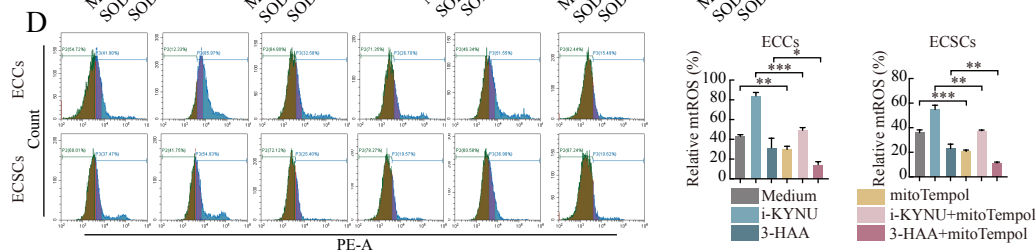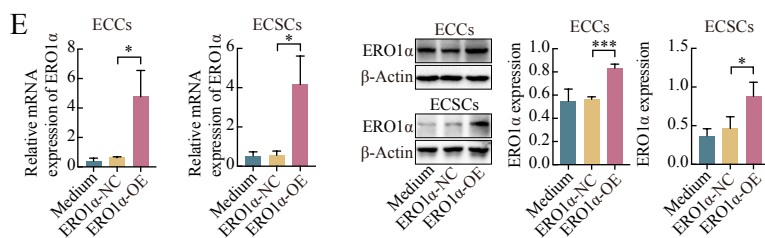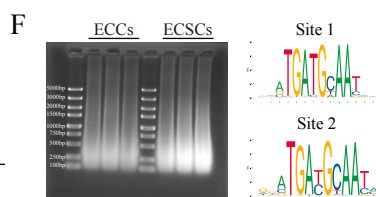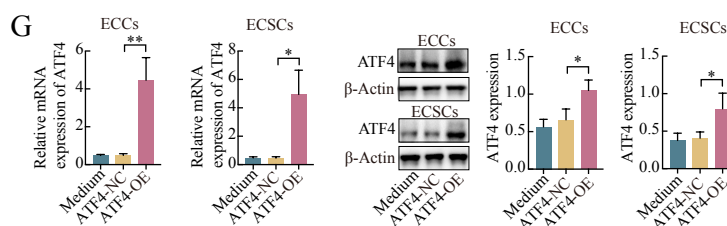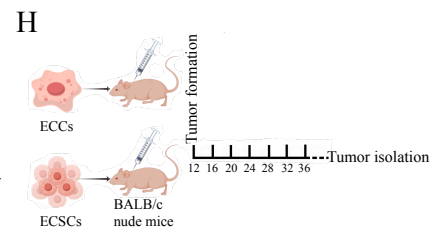

Supplement: Supplementary file 1 — Supplementary Material 1: Fig S1. A Flow cytometry results of macrophage sorting. CD86+ cells are identified as M1 macrophages, whereas CD163+ and CD86+CD163+ cells are referred to as M2 macrophages. B Seven peptide fragments of KYNU from the proteomic assay are shown. C The transfection efficiency of the SOD2 overexpression plasmid was verified by qRT‒PCR and western blot. D MitoTempol rescued the high level of mtROS caused by i-KYNU. Together with 3-HAA, mitoTempol mostly decreased the level of mtROS. E The transfection efficiency of the ERO1α overexpression plasmid was verified by qRT‒PCR and western blot. F Ultrasonic fragmentation in the ChIP assay was detected using agarose gel electrophoresis, and the motif logo is shown. G The transfection efficiency of the ATF4 overexpression plasmid was verified via qRT‒PCR and western blot. H Diagram showing the time of tumor formation in BALB/c (nu/nu) mice transplanted with control or treated cells. The data are presented as the means ± SDs (n = 3 per group), Student’s t test, one-way analysis of variance (ANOVA). *P< 0.05, **P < 0.01, ***P < 0.001 [file 13046_2025_3285_MOESM1_ESM.pdf]

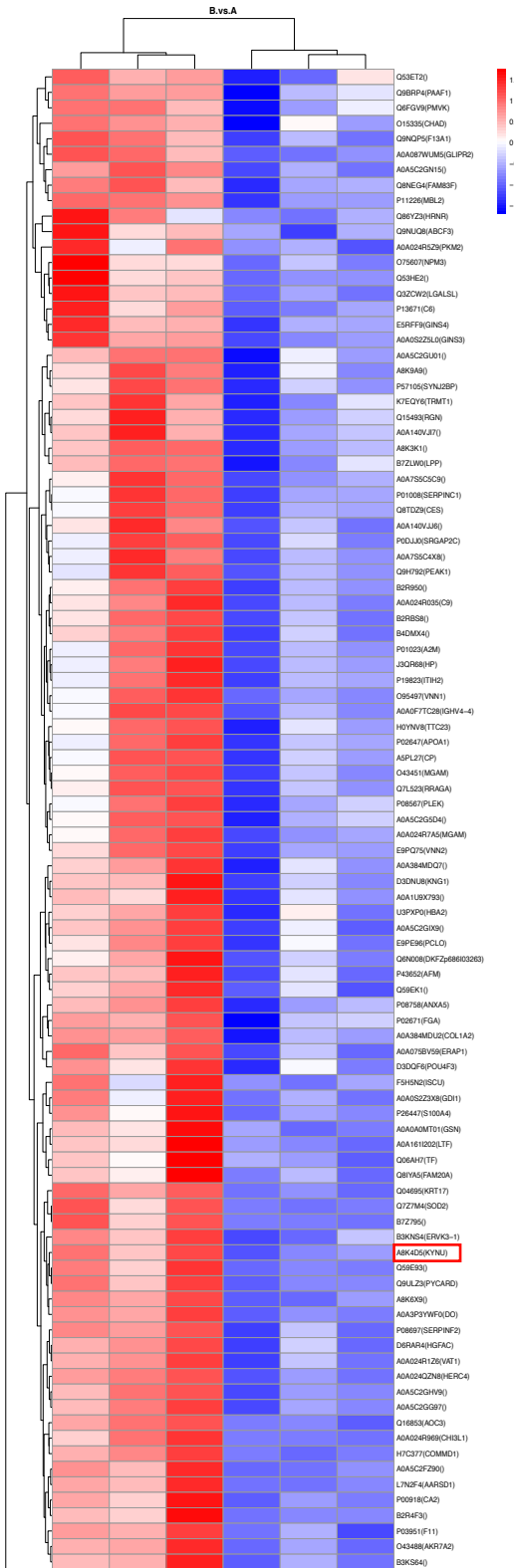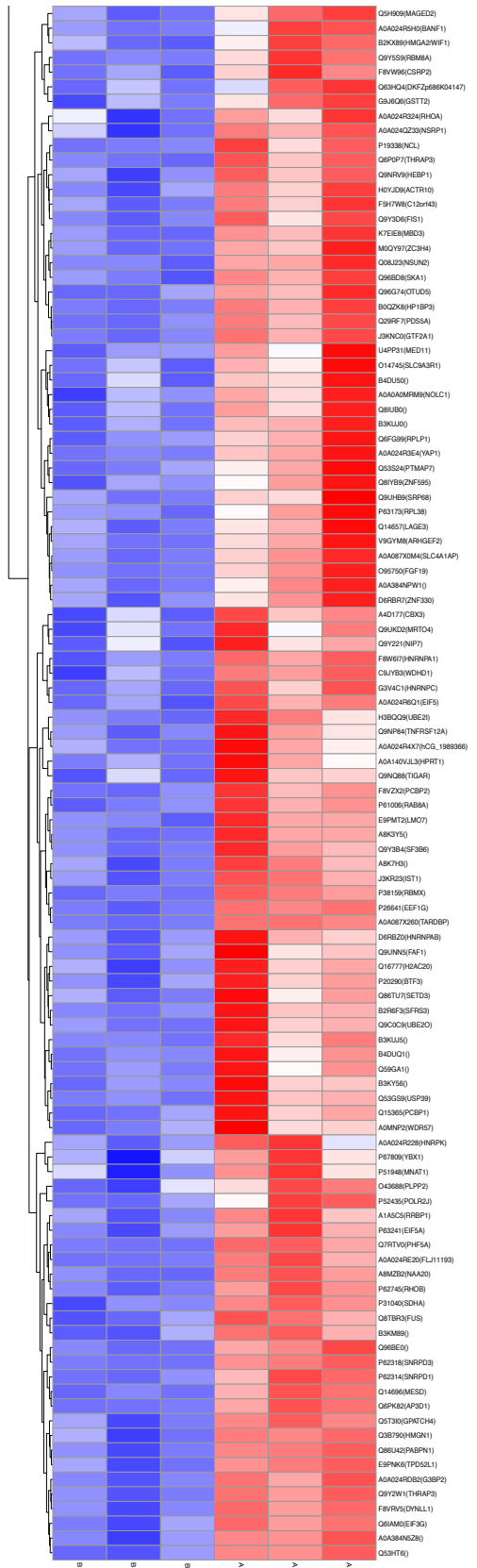

Supplement: Supplementary file 2 — Supplementary Material 2: Fig S2. The heatmap of the TMT Quantitative Proteomics Technical Services results. A total of 2,914 proteins were identified, with 97 highly expressed proteins and 106 proteins expressed at low levels. [file 13046_2025_3285_MOESM2_ESM.pdf]

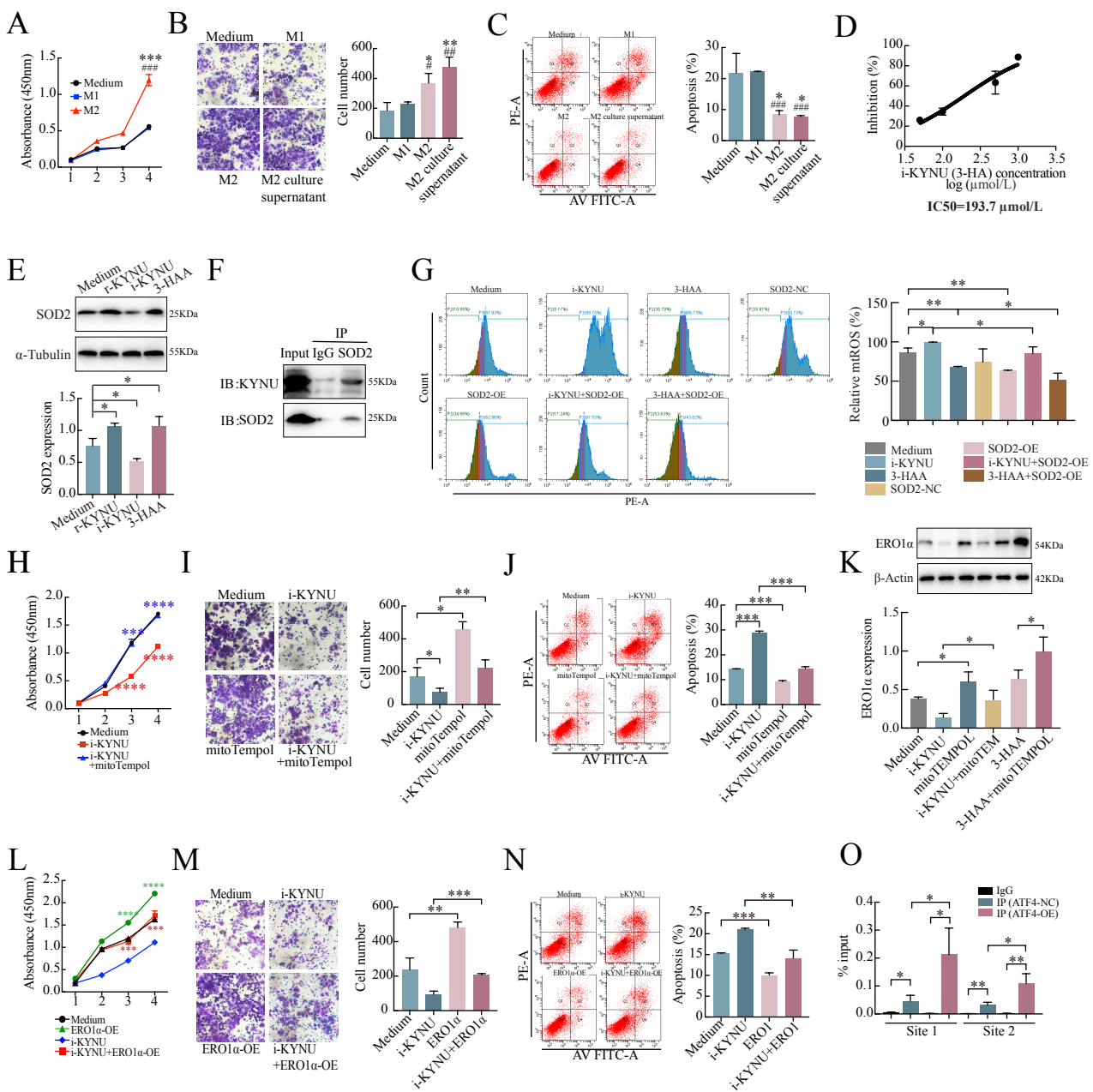

Supplement: Supplementary file 3 — Supplementary Material 3: Fig S3. Cell proliferative capacity A, cell invasion B, and cell apoptosis rate C of HEC-1A cells cocultured with M1 or M2 macrophages were detected by a CCK-8 assay, a transwell assay and flow cytometry. D IC50 values for the KYNU inhibitor in HEC-1A cells were detected by a CCK-8 assay. The concentrations used were 50 μmol/L, 100 μmol/L, 500 μmol/L, and 1,000 μmol/L. E The interaction between KYNU and SOD2 was verified by Co-IP. F The regulatory effects of the KYNU inhibitor, recombinant KYNU and 3-HAA on SOD2 were confirmed by western blot. G Flow cytometry analysis of the influence of KYNU, SOD2, MitoTempol and their combination on mtROS levels. The proliferative capacity H, invasion I, and apoptosis rate J of HEC-1A cells pretreated with the KYNU inhibitor or mitoTempol were detected by a CCK-8 assay, a transwell assay and flow cytometry. K western blot analysis of the influence of the KYNU inhibitors 3-HAA and mitoTempol as well as their combination on ERO1α expression. The cell proliferative capacity L, invasion M, and apoptosis rate N of the HEC-1A cells pretreated with the KYNU inhibitor or ERO1α-OE were detected via the CCK-8 assay, transwell assay and flow cytometry. O Two predicted binding sites of the KYNU promoter region with the transcription factor ATF4 were verified by a ChIP assay in the HEC-1A cell line. The data are presented as the means ± SDs (n = 3 per group), Student’s t test, one-way analysis of variance (ANOVA). *P < 0.05, **P < 0.01, ***P < 0.001. [file 13046_2025_3285_MOESM3_ESM.pdf]
