# Supplementary material for: M2 macrophage-secreted KYNU promotes stemness remodeling and malignant behavior in endometrial cancer via the SOD2-mtROS-ERO1α-UPRER axis
Source: J Exp Clin Cancer Res. 2025 Jul 4;44:193. doi: 10.1186/s13046-025-03285-y (PMC12231660; doi:10.1186/s13046-025-03285-y)
Supplement: Supplementary file 4 — Supplementary Material 4: Table S1. The sequences of primers used for qRT‒PCR and the binding sites in the promoter region of KYNU. Table S2. Information of the primary antibodies used in this study. Table S3. The plasmid sequences used in cell transfection. Table S4. Relationship of SOD2 expression with the clinical characteristics of patients with endometrial cancer. Pearson tests were used for statistical analysis. Table S5. Relationship of ERO1α expression with the clinical characteristics of patients with endometrial cancer. Pearson tests were used for statistical analysis. [file 13046_2025_3285_MOESM4_ESM.docx]

Table S1. The primer sequences of qRT-PCR and binding sites in the promoter region of KYNU.

| TNFα | | F: TGCTCCTCACCCACACCATCAG |
| --- | --- | --- |
|  |  | R: TCCCAAAGTAGACCTGCCCAGAC |
| HLA-DRα | | F: GCCGAGTTCTATCTGAATCCTGACC |
|  |  | R: GACCGTCTCCTTCTTTGCCATATCC |
| IL-6 | | F: GCCTTCGGTCCAGTTGCCTTC |
|  |  | R: GCCTCTTTGCTGCTTTCACACATG |
| iNOS | | F: TGCCCTGCTTTGTGCGGAATG |
|  |  | R: CTCCTCCTGGTAGATGTGGTCCTC |
| CD204 | | F: GACACTGATAGCTGCTCCGAATCTG |
|  |  | R: AAACACGAGGAGGTAAAGGGCAATC |
| CD301 | | F: CCTGCTGCTGGTCATCATCTGTG |
|  |  | R: TGGAGGCATTGTTGTTGAGAGTAGC |
| IL-10 | | F: GGGTTGCCAAGCCTTGTCTGAG |
|  |  | R: CCTTGATGTCTGGGTCTTGGTTCTC |
| CD206 | | F: TCCGACCCTTCCTTGACTAATCCTC |
|  |  | R: AGTATGTCTCCGCTTCATGCCATTG |
| SOD2 | | F: CCCGACCTGCCCTACGACTAC |
|  |  | R: AACGCCTCCTGGTACTTCTCCTC |
| KYNU | | F: ATGCGGATGATAAAGCCAAGAGAGG |
|  |  | R: TCAAAGCCAACATAACAACCCTTCG |
| ERO1 | | F: ACTGTGCTGTCAAACCATGTCAATC |
|  |  | R: TCAGAGATTCATCCACTGCTCCAAG |
| Primer for CHIP | Site 1 | F: TCCACAAGGGTTAAGTGAATTGC |
|  |  | R: CTAGGGATGAAGAATGTGCTTGAA |
|  | Site 2 | F: CCAGGCTAGTTTCCACTTGAAT |
|  |  | R: CCCTCCTTCAAATACACTGAGGA |

Table S2. Information of primary antibodies.

|  | Manufacturer | Dilution ratio |
| --- | --- | --- |
| CD133 | Proteintech, 66666-1-Ig, Wuhan, China | 1:5000 |
| CD44 | Proteintech, 60224-1-Ig, Wuhan, China | 1:5000 |
| SALL4 | Huaan, HA500482, Hangzhou, China | 1:1000 |
| SOX2 | Proteintech, 66411-1-Ig, Wuhan, China | 1:2000 |
| NANOG | Proteintech, 67255-1-Ig, Wuhan, China | 1:5000 |
| OCT4 | Proteintech, 60242-1-Ig, Wuhan, China | 1:5000 |
| KYNU | Thermo Fisher, MA5-29382, Waltham, USA | 1:800 |
| SOD2 | Proteintech, 66474-1-Ig, Wuhan, China | 1:10000 |
| ERO1 | Proteintech, 67416-1-Ig, Wuhan, China | 1:5000 |
| PERK | Proteintech, 24930-1-AP, Wuhan, China | 1:1000 |
| p-PERK | Affinity, DF7576, Melbourne, Australia | 1:1000 |
| EIF2α | Proteintech, 11170-1-AP, Wuhan, China | 1:5000 |
| p-EIF2α | Proteintech, 28740-1-AP, Wuhan, China | 1:2000 |
| ATF4 | Proteintech, 60035-1-AP, Wuhan, China | 1:2000 |
| α-Tubulin | Proteintech, 66031-1-Ig, Wuhan, China | 1:10000 |
| β-Actin | Proteintech, 66009-1-Ig, Wuhan, China | 1:10000 |

Table S3. The plasmid sequences used in cell transfection.

|  | Sequences |
| --- | --- |
| pcDNA3.1-SOD2 | atgttgagccgggcagtgtgcggcaccagcaggcagctggctccggttttggggtatctgggctccaggcagaagcacagcctccccgacctgccctacgactacggcgccctggaacctcacatcaacgcgcagatcatgcagctgcaccacagcaagcaccacgcggcctacgtgaacaacctgaacgtcaccgaggagaagtaccaggaggcgttggccaagggagatgttacagcccagatagctcttcagcctgcactgaagttcaatggtggtggtcatatcaatcatagcattttctggacaaacctcagccctaacggtggtggagaacccaaaggggagttgctggaagccatcaaacgtgactttggttcctttgacaagtttaaggagaagctgacggctgcatctgttggtgtccaaggctcaggttggggttggcttggtttcaataaggaacggggacacttacaaattgctgcttgtccaaatcaggatccactgcaaggaacaacaggccttattccactgctggggattgatgtgtgggagcacgcttactaccttcagtataaaaatgtcaggcctgattatctaaaagctatttggaatgtaatcaactgggagaatgtaactgaaagatacatggcttgcaaaaagtaa |
| pcDNA3.1-ERO1α | atgggccgcggctggggattcttgtttggcctcctgggcgccgtgtggctgctcagctcgggccacggagaggagcagcccccggagacagcggcacagaggtgcttctgccaggttagtggttacttggatgattgtacctgtgatgttgaaaccattgatagatttaataactacaggcttttcccaagactacaaaaacttcttgaaagtgactactttaggtattacaaggtaaacctgaagaggccgtgtcctttctggaatgacatcagccagtgtggaagaagggactgtgctgtcaaaccatgtcaatctgatgaagttcctgatggaattaaatctgcgagctacaagtattctgaagaagccaataatctcattgaagaatgtgaacaagctgaacgacttggagcagtggatgaatctctgagtgaggaaacacagaaggctgttcttcagtggaccaagcatgatgattcttcagataacttctgtgaagctgatgacattcagtcccctgaagctgaatatgtagatttgcttcttaatcctgagcgctacactggttacaagggaccagatgcttggaaaatatggaatgtcatctacgaagaaaactgttttaagccacagacaattaaaagacctttaaatcctttggcttctggtcaagggacaagtgaagagaacactttttacagttggctagaaggtctctgtgtagaaaaaagagcattctacagacttatatctggcctacatgcaagcattaatgtgcatttgagtgcaagatatcttttacaagagacctggttagaaaagaaatggggacacaacattacagaatttcaacagcgatttgatggaattttgactgaaggagaaggtccaagaaggcttaagaacttgtattttctctacttaatagaactaagggctttatccaaagtgttaccattcttcgagcgcccagattttcaactctttactggaaataaaattcaggatgaggaaaacaaaatgttacttctggaaatacttcatgaaatcaagtcatttcctttgcattttgatgagaattcattttttgctggggataaaaaagaagcacacaaactaaaggaggactttcgactgcattttagaaatatttcaagaattatggattgtgttggttgttttaaatgtcgtctgtggggaaagcttcagactcagggtttgggcactgctctgaagatcttattttctgagaaattgatagcaaatatgccagaaagtggacctagttatgaattccatctaaccagacaagaaatagtatcattattcaacgcatttggaagaatttctacaagtgtgaaagaattagaaaacttcaggaacttgttacagaatattcattaa |

Table S4. Relationship between SOD2 expression and the clinical characteristics of endometrial cancer.

| Clinical characteristics | N | Relative expression of SOD2 | P-values |
| --- | --- | --- | --- |
| Age | | | 0.1000 |
| ＜60 | 64 | 2.85±3.25 |  |
| ≥60 | 46 | 2.74±5.43 |  |
| FIGO stage | | | **<0.0001** |
| I-II | 71 | 1.43±1.73 |  |
| III-IV | 39 | 5.28±5.98 |  |
| Grade | | | 0.2762 |
| G1 | 49 | 2.08±2.62 |  |
| G2-3 | 61 | 3.36±5.12 |  |
| Invasion depth | | | **<0.0001** |
| Superficial | 82 | 1.16±1.45 |  |
| Deep | 28 | 7.58±5.81 |  |
| LVSI | | | 0.7396 |
| No | 85 | 2.89±4.52 |  |
| Yes | 25 | 2.46±3.10 |  |
| Lymphatic metastasis | | | 0.6571 |
| No | 83 | 2.99±4.57 |  |
| Yes | 27 | 2.20±2.92 |  |
| Distal metastasis | | | 0.8840 |
| No | 99 | 2.87±4.39 |  |
| Yes | 11 | 2.07±2.36 |  |

Table S5. Relationship between ERO1α expression and the clinical characteristics of endometrial cancer.

| Clinical characteristics | N | Relative expression of ERO1α | P-values |
| --- | --- | --- | --- |
| Age | | | 0.7925 |
| ＜60 | 64 | 1.93±2.06 |  |
| ≥60 | 46 | 2.58±3.43 |  |
| FIGO stage | | | **0.0001** |
| I-II | 71 | 1.41±1.14 |  |
| III-IV | 39 | 3.64±3.94 |  |
| Grade | | | 0.1692 |
| G1 | 49 | 1.72±1.39 |  |
| G2-3 | 61 | 2.59±3.40 |  |
| Invasion depth | | | 0.6805 |
| Superficial | 82 | 2.10±2.59 |  |
| Deep | 28 | 2.50±3.10 |  |
| LVSI | | | **0.0041** |
| No | 85 | 1.67±1.43 |  |
| Yes | 25 | 4.00±4.60 |  |
| Lymphatic metastasis | | | **0.0005** |
| No | 83 | 1.56±1.32 |  |
| Yes | 27 | 4.17±4.47 |  |
| Distal metastasis | | | **0.0008** |
| No | 99 | 1.66±1.34 |  |
| Yes | 11 | 7.12±5.63 |  |
